# Statistical inference after variable selection in Cox models: a neutral simulation study

**DOI:** 10.1186/s12874-026-02887-0

**Published:** 2026-06-23

**Authors:** Lena Schemet, Sarah Friedrich-Welz

**Affiliations:** 1 Department of Mathematics, University of Augsburg, Augsburg, Bavaria 86159 Germany; 2 Center for Advanced Analytics and Predictive Sciences (CAAPS), University of Augsburg, Augsburg, Bavaria 86159 Germany

**Keywords:** Survival analysis, Cox model, Lasso, Post-selection inference, Debiased Lasso

## Abstract

Choosing relevant predictors is central to the analysis of biomedical time-to-event data. Classical frequentist inference, however, presumes that the set of covariates is fixed in advance and does not account for data-driven variable selection. As a consequence, naive post-selection inference may be biased and misleading. In right-censored survival settings, these issues may be further exacerbated by the additional uncertainty induced by censoring. We investigate several inference procedures applied after variable selection for the coefficients of the Lasso and its extension, the adaptive Lasso, in the context of the Cox model. The methods considered include sample splitting, post-selection inference procedures that condition explicitly on the Lasso selection event, and the debiased Lasso. Because these methods address different inferential targets, we distinguish selected-submodel targets from full-model targets and interpret empirical coverage, interval width, power, and type I error accordingly. Their performance is examined in a neutral simulation study reflecting realistic covariate structures and censoring rates commonly encountered in biomedical applications. The primary focus is post-selection inference after Cox-Lasso variable selection, not a comprehensive benchmark of very-high-dimensional variable-selection performance. To complement the simulation results, we illustrate the practical behavior of these procedures in an applied example using a publicly available survival dataset.

**Supplementary Information:**

The online version contains supplementary material available at 10.1186/s12874-026-02887-0.

## Introduction

Variable selection has become an integral component of modern regression analysis, particularly in biomedical research where complex covariate structures and limited sample sizes are common. Methods such as the Lasso [1] and its variants [2] are widely used to identify relevant predictors while performing regularization to stabilize estimation. However, classical inferential procedures are not designed to accommodate the data-driven nature of variable selection. As a consequence, naive confidence intervals and *p*-values computed after model selection may be severely biased, anticonservative, and ultimately misleading [3].

This problem is well known in linear and generalized linear models, and a growing body of work has highlighted the need for inference procedures that explicitly acknowledge the selection step [4]. In the context of time-to-event outcomes the issue is even more pronounced. Right censoring introduces additional uncertainty, and the dependence of event times on both covariates and censoring mechanisms complicates the use of standard asymptotic arguments [5]. Despite the widespread use of penalized Cox models in biomedical applications [6, 7] principled inference for their coefficients after variable selection remains challenging and comparatively underexplored.

Several methodological proposals have been developed to address these limitations. Sample splitting offers a conceptual toy strategy by separating model selection from subsequent inference. Its effectiveness typically relies on sufficiently large sample sizes, since both steps are carried out on only a subset of the available data which may reduce statistical efficiency [8]. Post-selection inference conditions explicitly on the selected model and can yield valid post-selection confidence intervals in regression settings. General frameworks for valid and exact post-selection inference have been developed and theoretically justified primarily for linear regression models [3, 4, 9]. Extensions of these approaches to the Cox model are currently limited [10, 11]. In particular, exact post-selection inference procedures for Cox regression have so far been studied only under fixed shrinkage and without randomization. Randomized post-selection inference has been shown to improve power and stability in linear models [12, 13], but corresponding theory and implementations are not yet established for the Cox model.

In addition to post-selection inference methods that explicitly condition on the selection event, there exist debiasing approaches for inference after variable selection. These methods apply approximate one-step corrections to penalized estimators in order to restore asymptotic normality. As a result, inference can be carried out after variable selection without explicitly conditioning on the selected model. Their theoretical properties and empirical performance have been predominantly investigated for linear regression models [14, 15]. Consequently, despite substantial progress in low- and high-dimensional linear models, the available evidence for the validity and practical performance of inference after variable selection in survival analysis remains comparatively sparse [10, 11].

Motivated by these considerations, we systematically evaluate inference procedures applied after variable selection for Lasso-type estimators in Cox models. Building on the framework of Kammer et al. [16], who assessed inference after variable selection in Gaussian settings, we extend their simulation design to right-censored survival data and investigate how censoring, covariate dependence, and model sparsity affect the validity and efficiency of competing approaches. The methods examined include sample splitting, exact post-selection inference, and the debiased Lasso. We place particular emphasis on two issues raised by such comparisons: the method-specific inferential target and the validity conditions under which each interval should be interpreted.

Our main contributions are:

- We adapt and extend the simulation study of Kammer et al. [16] to the Cox model, incorporating realistic survival and censoring structures relevant to biomedical applications.
- We provide a neutral comparative evaluation of sample splitting, exact post-selection inference, and the debiased Lasso, focusing on method-specific coverage, confidence-interval width, and power under a wide range of simulation scenarios.
- We explicitly distinguish selected-submodel targets from full-model targets and interpret all inferential performance measures relative to the target addressed by each method.
- We illustrate the practical implications of these methods in an applied example using publicly available survival data.

The remainder of the manuscript is organized as follows. The Methods section introduces the Cox model setting, the Lasso and adaptive Lasso estimators, and the inference procedures considered, including sample splitting, post-selection inference, and debiasing approaches. The Simulation study section describes the simulation study design, including data-generating mechanisms, estimands, and performance measures. The Results section presents the simulation results. The Real data example section illustrates the proposed methods using a real data example. The Discussion section concludes with a discussion of the main findings and implications.

Overall, our results offer guidance for practitioners seeking reliable inference after Lasso-based variable selection in survival analysis and highlight methodological aspects that warrant further research.

## Methods

This section describes the methodological framework of the study. We first introduce the model setting and notation after which we present the variable selection procedures and post-selection inference methods considered.

### Setting

Let $X = (X^{(1)}, \ldots , X^{(p)})^\top$ denote a *p*-dimensional covariate vector, with $(\cdot )^\top$ denoting the transpose of any vector of interest, and let *T* denote the failure time of interest.

Under right censoring with censoring time *C*, we observe the survival time $Y = \min (T, C)$ and the event indicator $\delta = \mathbb {1}(T \le C)$. We assume non-informative censoring in the sense of Andersen et al. [5], that is, the censoring mechanism is independent of the event process conditional on the observed covariates and does not depend on the unknown regression parameters.

For *n* independent individuals, we observe i.i.d. data $(Y_i, \delta _i, X_i)$ for $i = 1, \ldots , n$, where $X_i = (X_i^{(1)}, \ldots , X_i^{(p)})^\top$ denotes the *p*-dimensional covariate vector.

The Cox model [17] specifies the conditional hazard at time *t* as

$$\begin{aligned} h(t \mid X) = h_0(t) \exp (X^\top \beta ^0), \end{aligned}$$

where $h_0(t)$ is the unknown baseline hazard function and $\beta ^0 = (\beta _1^0, \ldots , \beta _p^0)^\top$ denotes the unknown true vector of regression coefficients.

Given the observed data the log partial likelihood, viewed as a function of a parameter $\beta \in \mathbb {R}^p$, is given by

1 $$\begin{aligned} \ell (\beta ) = \sum \limits _{i=1}^n \delta _i \left( X_i^\top \beta - \log \sum \limits _{j : Y_j \ge Y_i} \exp (X_j^\top \beta ) \right) . \end{aligned}$$

Estimation of the true regression parameter $\beta ^0$ is based on maximizing the log partial likelihood (1), yielding an estimator $\hat{\beta }$. In settings with many covariates, penalized approaches such as the Lasso and its variants have become standard tools [6, 7, 18].

### Lasso

The Lasso estimator [1], adapted to the Cox model, is obtained by maximizing the $\ell _1$-penalized partial likelihood,

$$\begin{aligned} L_P(\beta ) = \ell (\beta ) - \lambda \sum \limits _{j=1}^p |\beta _j|, \end{aligned}$$

where $\lambda> 0$ is the regularization parameter.

The Lasso has several attractive properties [7]. In particular, the $\ell _1$-penalty performs variable selection by shrinking some coefficients exactly to zero. The method has become widely used in linear regression and increasingly in Cox models, especially in biomedical applications involving large-scale covariate data [19, 20].

However, these advantages come with well-known limitations:

1. The Lasso tends to include too many variables, leading to an inflated false-positive rate in variable selection [21].
2. Estimated coefficients are biased toward zero, especially for variables with larger true effects [14, 22].

One approach to mitigate this shrinkage bias is the adaptive Lasso [2], which incorporates data-dependent weights $w_j$, $j \in \{1,\ldots ,p\}$, into the penalty term. A common choice is to define these weights as inverse functions of preliminary coefficient estimates, for example $w_j = 1 / |\hat{\beta }_j|^\gamma$ with $\gamma \in \mathbb {N}$. This weighting scheme reduces shrinkage for larger coefficients while preserving sparsity among smaller ones. Under this choice, the adaptive Lasso estimator achieves $\sqrt{n}$-consistency and satisfies the oracle property [7].

Despite these improvements, adaptive Lasso procedures do not always achieve satisfactory coverage properties in inference after variable selection for linear regression [3]. Motivated by this, we consider and compare both the standard Lasso and the adaptive Lasso in the Cox model setting.

### Conceptual framework: inference after variable selection

Post-selection inference addresses inferential questions that arise when hypotheses, models, or inferential targets are not specified prior to observing the data, but are generated as part of the data-analytic process itself [3, 9]. This setting naturally arises in variable selection problems, where the data are first used to choose a subset of covariates and inference is subsequently reported only for the selected variables.

In the context of variable selection, the data are used to select a submodel $M \subseteq M_F$, where $M_F$ denotes the full set of candidate predictors, for example via the Lasso or adaptive Lasso. Inference is then performed for regression coefficients associated with the selected variables. Classical frequentist inference, however, constructs $(1-\alpha )$ confidence intervals for full-model parameters $\beta _j^F$ such that

$$\begin{aligned} \mathbb {P}\!\left( \beta _j^F \in CI_j \right) = 1-\alpha , \end{aligned}$$

under the assumption that the inferential procedure is independent of any data-driven model choice. Once variable selection is introduced, these guarantees generally fail, because the selection step alters the distribution of estimators and test statistics [23, 24].

#### Full-model and selected-submodel targets

Variable selection changes not only the distribution of the estimators, but also the inferential target for some post-selection procedures. For submodel-based procedures, inference is conducted in the model induced by the selection procedure rather than in the full candidate model. In this selected-submodel view, regression coefficients are defined relative to the selected set of variables and generally need not coincide with their full-model counterparts.

For a given submodel $M \subseteq M_F$, the associated population-level coefficient vector is

$$\begin{aligned} \beta _{M} = (\beta _{j,M})_{j \in M} = \beta _M(\beta ^F), \end{aligned}$$

which can be viewed as the projection of the full-model parameter $\beta ^F$ onto the submodel corresponding to *M* [4, 12]. In linear regression, these submodel coefficients admit closed-form expressions, whereas in the Cox model no such analytic representation is available [5, 25]. Instead, they are implicitly defined through the partial likelihood and obtained numerically, for example via Newton–Raphson-type algorithms [5, 17].

This distinction is particularly important in Cox regression because regression coefficients are generally non-collapsible: omitting covariates can change the coefficient associated with a retained variable, even in the absence of classical confounding [26–28]. Consequently, full-model coefficients $\beta _j^F$ and selected-submodel coefficients $\beta _{j,M}$ should not be interpreted as the same estimand.

#### Post-selection confidence intervals as primary objects of evaluation

The inferential targets considered in this study are method-specific. However, from an applied perspective, all investigated procedures are used for the same practical purpose: after a data-driven variable selection step, confidence intervals are reported for the selected variables.

We therefore use the term *post-selection confidence interval* (PSCI) in an operational sense to denote a confidence interval reported after variable selection. This terminology does not imply that all intervals share the same formal inferential target or the same validity guarantee. The parameters covered by these intervals are the estimands, whereas the intervals themselves are the primary objects of empirical evaluation in the simulation study.

For sample splitting and exact conditional post-selection inference, PSCIs are interpreted as intervals for selected-submodel coefficients $\beta _{j,\widehat{M}}$, where $\widehat{M}$ denotes the selected model. These methods therefore address inference conditional on, or separated from, the selection step and are evaluated relative to the selected-submodel target.

For a fixed selected model *M* and a selected variable $j \in M$, the corresponding conditional coverage statement can be written conceptually as

2 $$\begin{aligned} \mathbb {P}\!\left( \left. \beta _{j,M} \in PSCI_{j,M}\;\right| \;\widehat{M}=M\right) \approx 1-\alpha . \end{aligned}$$

For sample splitting, this validity relies on the independence between the selection and inference samples and on the large-sample validity of the Cox model fitted in the inference sample. For exact conditional PSI, it relies on conditioning on the selection event, including the selected model and, in the implementation considered here, a fixed penalty parameter.

In contrast, the debiased Lasso targets full-model coefficients $\beta _j^F$ under high-dimensional regularity conditions and does not condition on the selection event. When debiased Lasso intervals are reported only for variables selected by the preceding Lasso step, their empirical performance is therefore assessed as full-model coverage among selected variables. This should not be interpreted as a conditional selected-submodel coverage guarantee.

For the debiased Lasso, the reported interval for a selected variable *j* has the form

3 $$\begin{aligned} PSCI^{\textrm{DB}}_j = \left[ \tilde{\beta }_j - z_{1-\alpha /2}\frac{\widehat{\sigma }_j}{\sqrt{n}},\, \tilde{\beta }_j + z_{1-\alpha /2}\frac{\widehat{\sigma }_j}{\sqrt{n}}\right] , \end{aligned}$$

where $\tilde{\beta }_j$ denotes the debiased estimator and $\widehat{\sigma }_j$ its estimated asymptotic standard deviation. At the empirical evaluation stage, if *S* denotes the set of simulation repetitions, these intervals are evaluated against the full-model target $\beta _j^F$ among variables selected by the preceding Lasso step:

4 $$\begin{aligned} \widehat{\textrm{Cov}}_{\textrm{DB}} = \frac{\sum \nolimits _{s \in S}\sum \nolimits _{j \in \widehat{M}_s}\textbf{1}\!\left\{ \beta _j^F \in PSCI^{\textrm{DB}}_{j,s}\right\} }{\sum \nolimits _{s \in S} |\widehat{M}_s|}. \end{aligned}$$

This empirical quantity differs from the conditional selected-submodel coverage criterion in Eq. (2).

Consequently, throughout the manuscript, coverage, confidence-interval width, power, and type I error are interpreted relative to the method-specific target. Direct comparisons across methods are understood as comparisons of practically reported intervals after selection, not as evidence that all methods estimate the same parameter or satisfy the same formal validity statement.

### Inference procedures

We now describe the specific inference procedures considered in this study. All methods are applied after Lasso variable selection and are used to construct post-selection confidence intervals, but they differ in their inferential targets, conditioning strategies, and coverage guarantees.

#### Sample splitting

The separation of model selection and statistical inference was first discussed by Cox [8]. Sample splitting translates this idea into a practical procedure by dividing the data into two disjoint parts.

In the first step, one part of the data is used to select a submodel $\widehat{M}$. In the second step, the remaining data are used to estimate the corresponding submodel coefficients and to construct PSCIs. Since inference is performed using data that was not involved in the selection step, the resulting PSCIs are interpreted relative to the conditional selected-submodel coverage property (2). PSCIs are obtained by fitting an unpenalized Cox model to the selected variables using the inference subsample and constructing standard Wald-type intervals based on the partial likelihood.

In applied work, a simple 50/50 split of the data is frequently used as a default choice. This practice is supported by simulation studies in linear regression settings, which suggest that such a split can provide a reasonable compromise between stable model selection and efficient inference [12, 29]. However, no comparable guidance is currently available for the Cox model, where the effective information content depends on the censoring mechanism and the sample size [10, 11]. By choosing the split proportion, one can balance selection accuracy and inferential precision. For simplicity, we use a 50/50 split throughout. From an implementation perspective, sample splitting is straightforward and computationally feasible. It does not require constrained optimization or matrix inversions beyond those encountered in standard unpenalized Cox model fitting.

A natural alternative to a single split is multiple data splitting, in which the selection–inference split is repeated several times and the resulting inferential evidence is aggregated across repetitions [30]. Such procedures can reduce the dependence of the results on one arbitrary random split and may improve stability and power. At an algorithmic level, an analogous approach could be combined with Cox regression by repeating Lasso-based selection on one part of the data, refitting an unpenalized Cox model on the held-out part, and aggregating the resulting split-specific evidence. We do not include multiple splitting as a separate comparator in the present simulation study, because our aim was to evaluate a small set of directly available and commonly used post-selection inference strategies; see [30] for the general multiple-splitting framework.

#### Exact conditional post-selection inference

The exact conditional post-selection inference framework (exact PSI) introduced by Lee et al. [4] provides finite-sample valid inference after Lasso selection by conditioning on the selection event.

Inference is carried out conditional on the selected submodel and targets the corresponding submodel-specific estimands. As a consequence, the resulting PSCIs are interpreted relative to the conditional selected-submodel coverage property (2).

For the Cox model adaptations are required due to the structure of the partial likelihood, but the underlying conditional inference principle remains conceptually unchanged. Available software implementations allow exact conditional post-selection inference when the penalty parameter $\lambda$ is treated as fixed.

Accordingly, exact PSI is applicable when the regularization parameter is specified in advance rather than chosen in a data-dependent or prediction-oriented manner. For linear regression models, randomized extensions of exact PSI have been proposed to accommodate data-driven tuning parameter selection. Such randomized procedures are currently unavailable for the Cox model and are computationally demanding even in the linear setting [16].

#### Debiased Lasso

The debiased Lasso was originally proposed for linear regression models to enable valid statistical inference by correcting the shrinkage bias induced by $\ell _1$ penalization [14, 15]. The approach was subsequently extended to the Cox proportional hazards model by Lu and Xia [31].

The central idea is to augment the Lasso estimator with a one-step correction that removes the leading bias term and thereby restores asymptotic normality. For the Cox model, this is achieved by replacing the least-squares score function and information matrix with their counterparts derived from the partial likelihood. Inference is then based on the score function of the Cox partial likelihood and an approximation of the inverse Fisher information matrix that accounts for censoring.

Specifically, the debiased estimator for coefficient *j* is given by

$$\begin{aligned} \tilde{\beta }_j = \widehat{\beta }_j + \widehat{\Theta }_j^\top U(\widehat{\beta }), \end{aligned}$$

where $\widehat{\beta }_j$ is the *j*th component of the Lasso estimator, $U(\widehat{\beta })$ denotes the score function of the Cox partial likelihood evaluated at $\widehat{\beta }$, and $\widehat{\Theta }_j$ denotes the *j*th row of an estimator of the inverse Fisher information matrix [31].

Under suitable regularity conditions, the debiased estimator $\tilde{\beta }_j$ is asymptotically normal, which enables the construction of debiased PSCIs of the form

$$\begin{aligned} PSCI^{\textrm{DB}}_j = \tilde{\beta }_j \pm z_{1-\alpha /2}\, \widehat{\sigma }_j / \sqrt{n}, \end{aligned}$$

where $z_{1-\alpha /2}$ denotes the $(1-\alpha /2)$-quantile of the standard normal distribution and $\widehat{\sigma }_j$ is a consistent estimator of the asymptotic standard deviation of $\tilde{\beta }_j$. For the Cox model, $\widehat{\sigma }_j$ is obtained from an approximation of the inverse Fisher information matrix $\widehat{\Theta }_j$ based on nodewise regression, which accounts for censoring and avoids direct inversion in high dimensions [15, 31].

Unlike sample splitting and exact PSI, the debiased Lasso targets the full-model estimand $\beta _j^F$ (or its partial-likelihood projection) and does not condition on the selection event. It therefore performs inference after variable selection without explicitly accounting for the selection step.

From a computational perspective, the debiased Lasso is more demanding than sample splitting and exact PSI, as it requires fitting multiple auxiliary regression models to approximate the inverse Fisher information matrix [15]. An R implementation for debiased inference in the Cox model is available as research code by Lu and Xia [31].

## Simulation study

We conducted a comprehensive simulation study to compare inference procedures applied after variable selection in Cox models. The study is designed to systematically assess how different post-selection inference approaches perform under varying sample sizes, covariate dimensions, correlation structures, censoring levels, and tuning strategies. Particular emphasis is placed on post-selection inferential validity, estimation efficiency, and the relationship between inferential and predictive performance.

The simulation design and reporting follow the ADEMP structure [32].

### Aims (A)

The aim of this simulation study was to evaluate post-selection inference procedures for moderate-dimensional Cox regression settings after data-driven variable selection. We examined the coverage of the resulting confidence intervals, together with the validity of the associated method-specific hypotheses, the correctness and stability of the selected submodels, and the resulting prediction performance. All procedures were compared across a broad range of data-generating mechanisms reflecting typical biomedical survival settings.

### Data-generating mechanisms (D)

We conducted a Monte Carlo simulation study with $n_{\text {sim}}=1000$ repetitions per scenario. We denote the set of simulation iterations by $S=\{1,\ldots ,n_{\text {sim}}\}$. Sample sizes *n* ranged from 75 to 800, starting at $n=75$ and increasing in steps of 100 thereafter (Table 2).

#### Toy simulation

For each individual, we generated a *p*-dimensional covariate vector with $p \in \{10, 20, 50\}$. Covariates were drawn from a multivariate normal distribution with zero mean and correlation structure

$$\begin{aligned} \Sigma _{ij} = \rho ^{|i-j|},\qquad \rho \in \{0.0, 0.3\}, \end{aligned}$$

yielding either independent or moderately correlated predictors. In scenarios designed to mimic mixed data types, a subset of covariates was dichotomized to obtain approximately Bernoulli(0.5) variables.

The true coefficient vector $\beta ^0$ was generated according to one of four predefined patterns (Table 1): An all-ones pattern with equal non-zero effects across all covariates, a high-contrast pattern featuring alternating large and small effects in the first four coefficients, a realistic pattern with moderate effects on a limited number of covariates, and a sparse pattern with only one or two active coefficients. All remaining coefficients were set to zero, depending on the dimension *p*.

**Table 1:** Coefficient patterns used for the data-generating vector$\beta ^0$

	Coefficient pattern	Description
allones	(1, 1, . . . , 1)	Equal effects on all covariates.
highcontrast	(0.3, 1.0, 0.3, 1.0, 0, . . . , 0)	Alternating large and small effects.
realistic	(0.8, 0.7, 0.5, 0.8, 0, . . . , 0)	Moderate effects on a few covariates.
sparse	(1, 1, 0, . . . , 0)	Two active coefficients.

The simulation grid was defined by the design factors listed in Table 2. In the toy simulations, each scenario was evaluated under two baseline hazard distributions (exponential and Weibull) and four coefficient patterns (all-ones, high-contrast, realistic, and sparse). In contrast, the METABRIC simulation is based on a single Weibull baseline hazard and an empirically calibrated coefficient pattern derived from the METABRIC data. All remaining design factors, including sample size and target censoring proportion, were varied analogously to the toy settings.

**Table 2:** Design factors varied in the simulation study

Factor	Toy simulations	Realistic simulations (METABRIC)
Sample size *n*	75–800	75–800
Number of covariates *p*	10, 20, 50	10
Correlation $\rho$	0.0, 0.3	—
Target censoring proportion	0, 0.10, 0.30	0, 0.10, 0.30
Baseline distribution	Exponential, Weibull	Weibull
Coefficient pattern	four different settings (Table 1)	real-data–based pattern

Event times were generated from a Cox model with linear predictor $X^\top \beta ^0$ based on Ramos et al. [33]. Two baseline hazard distributions were considered. For the exponential baseline,

$$\begin{aligned} T = -\frac{\log U}{\exp (X^\top \beta ^0)},\qquad U \sim \textrm{Unif}(0,1), \end{aligned}$$

which corresponds to a constant baseline hazard. For the Weibull baseline, survival times were generated by inverse transform sampling as

$$\begin{aligned} T=\left( \frac{-\log U}{s\,\exp (X^\top \beta ^0)}\right) ^{1/k},\qquad U\sim \textrm{Unif}(0,1), \end{aligned}$$

which corresponds to a Cox model with cumulative baseline hazard $H_0(t)=s\,t^{k}$ and baseline hazard $h_0(t)=s\,k\,t^{k-1}$. Here, $k>0$ denotes the Weibull shape parameter, yielding an increasing baseline hazard for $k>1$ and a decreasing baseline hazard for $k<1$, while $s>0$ is a scale parameter controlling the overall timescale of the baseline hazard.

In all Weibull scenarios, we fixed the scale parameter to $s=1$ to simplify the simulation design. Since the baseline scale is not identifiable in the Cox model and does not affect the partial likelihood for the regression coefficients, this choice is not expected to influence inference on $\beta$ and allows us to focus on post-selection inference properties [25, 34]. Moreover, the shape parameter was fixed to $k=2$, corresponding to a moderately increasing baseline hazard. Varying the baseline hazard shape was not a primary focus of this study, as inference after variable selection in Cox models is expected to primarily depend on the regression structure rather than on the specific parametric form of the baseline hazard.

Independent censoring was imposed through a simple administrative censoring mechanism. For each replicate, we first generated event times $\{T_i\}_{i=1}^n$ and then defined a censoring cutoff $c^\star$ as the $(1-\pi _C)$-quantile of $\{T_i\}_{i=1}^n$, where $\pi _C \in \{0,0.1,0.3\}$ denotes the target censoring proportion. Observed times were defined as $Y=\min (T,c^\star )$ with event indicator $\delta =\textbf{1}(T \le c^\star )$. This construction yields censoring proportions close to the target level by design [33].

To avoid ties, small deterministic jitters were added to duplicated event (and, where applicable, censoring) times only. Specifically, for each group of identical times, we added offsets of order $\varepsilon _T=10^{-8}$ to the event times and $\varepsilon _C=5\cdot 10^{-9}$ to the censoring times, scaled by the range of the corresponding time variable.

#### Realistic simulation

In addition to the toy settings, we considered a realistic simulation scenario calibrated to the Molecular Taxonomy of Breast Cancer International Consortium (METABRIC) study [35], a large breast cancer cohort with long-term clinical follow-up. The same cohort is also used for the real-data illustration in the Real data example section. The aim of this design is to retain full control over the data-generating mechanism while incorporating empirically motivated covariate distributions, effect sizes, and dependence structures. This approach follows the principles of realistic parametric simulation designs discussed by Sauer et al. [36].

We constructed a fixed clinical covariate set reflecting the variables used in the real data analysis in the Real data example section, including age at diagnosis, tumor stage, ER/HER2 status, progesterone receptor status, chemotherapy, hormone therapy, radiotherapy, histologic grade, tumor size, number of positive lymph nodes, and the Nottingham Prognostic Index. Categorical predictors were encoded via an explicit dummy design matrix without intercept. To obtain a stable and reproducible design across simulation replicates, constant columns and very rare 0/1 dummy variables were removed, and all remaining covariates were centered and scaled. The resulting stabilized design matrix was kept fixed throughout the simulation study.

Event times were generated from a Cox model with a Weibull baseline hazard using inverse transform sampling, following the same data-generating mechanism as in the toy simulation. The Weibull baseline was chosen because it provides a flexible monotone hazard function and, in preliminary experiments comparing several parametric baseline distributions (including exponential, log-normal, and log-logistic), yielded the best discrimination performance in terms of time-dependent AUC. Details of this model comparison are provided in the Supplementary Material S1.1.

A Weibull model was fitted once to the stabilized METABRIC design matrix, and the fitted regression coefficients were treated as the simulation truth. In each simulation replicate, covariate profiles were resampled with replacement from the METABRIC cohort, and event times were generated from the calibrated Weibull baseline model. Target censoring proportions were imposed using the same administrative censoring mechanism as in the toy simulation, with censoring levels varied across scenarios.

Overall, the METABRIC simulation represents a structured extension of the toy design: the survival time generation, baseline hazard formulation, and censoring mechanism are identical, while the covariate distribution and regression coefficients are grounded in real clinical data. This allows us to study post-selection inference procedures under controlled yet realistically calibrated conditions.

#### Design factors

The true coefficient vector $\beta ^0$ was generated according to one of four predefined patterns (Table 1): An all-ones pattern with equal non-zero effects across all covariates, a high-contrast pattern featuring alternating large and small effects in the first four coefficients, a realistic pattern with moderate effects on a limited number of covariates, and a sparse pattern with only one or two active coefficients. All remaining coefficients were set to zero, depending on the dimension *p*.

The primary simulation grid was designed to study post-selection inference after Cox-Lasso variable selection in moderate-dimensional survival settings, not as a benchmark of very-high-dimensional Lasso screening.

To address the dimensionality concern raised during review, we additionally report a small exploratory $p=100$ sensitivity analysis in the Supplementary Material. These runs are not part of the primary simulation grid and are interpreted only descriptively, because the sparse signal structure makes variable screening a more dominant component of the observed operating characteristics.

### Estimands (E)

The simulation study distinguishes between the parameters targeted by each method and the confidence intervals reported after variable selection. The target parameters are the estimands, whereas the post-selection confidence intervals (PSCIs) and their associated rejection decisions are the primary empirical objects of evaluation.

In simulation iteration $s \in S$, let $\widehat{M}_s \subseteq M_F$ denote the model selected by the Lasso-based procedure. For submodel-based procedures, including naive refitting, sample splitting, and exact PSI, inference is interpreted relative to the selected-submodel coefficient $\beta _{j,\widehat{M}_s}$ for $j\in \widehat{M}_s$. For the debiased Lasso, inference is interpreted relative to the corresponding full-model coefficient $\beta _j^F$ and is evaluated among variables selected by the preceding Lasso step.

Thus, the same empirical reporting rule—constructing and displaying intervals only for selected variables—can correspond to different formal inferential targets. This distinction is central for interpreting coverage, interval width, power, and type I error in the results below. Table 3 summarizes the target and coverage interpretation for each method.

**Table 3:** Inferential targets and coverage interpretation of the investigated methods

Method	Target	Conditioning principle	Coverage
Full Cox model	Full-model coefficient$\beta _j^F$;$H_{0,j}^F:\beta _j^F=0$	No selection	Standard full-model Wald coverage
Oracle Cox model	Oracle-submodel coefficient$\beta _{j,M_0}$;$H_{0,j,M_0}:\beta _{j,M_0}=0$	True active set known	Benchmark coverage under known model
Naive refit/refit0	Selected-submodel coefficient$\beta _{j,\widehat{M}}$;$H_{0,j,\widehat{M}}:\beta _{j,\widehat{M}}=0$	No explicit adjustment for selection	Post-selection refit coverage; does not formally adjust for selection
Sample splitting	Selected-submodel coefficient$\beta _{j,\widehat{M}}$;$H_{0,j,\widehat{M}}:\beta _{j,\widehat{M}}=0$	Selection and inference performed on independent data splits	Conditional selected-submodel coverage based on the inference sample
Exact PSI	Selected-submodel coefficient$\beta _{j,\widehat{M}}$;$H_{0,j,\widehat{M}}:\beta _{j,\widehat{M}}=0$	Conditional on the Lasso selection event; fixed$\lambda$in the formal guarantee	Conditional post-selection coverage under the implemented selection event
Debiased Lasso	Full-model coefficient$\beta _j^F$;$H_{0,j}^F:\beta _j^F=0$	No explicit conditioning on selection	Asymptotic full-model coverage, evaluated among selected variables

Secondary estimands include the associated method-specific null hypotheses and the resulting after-selection models. For submodel-based procedures, power and type I error are evaluated for the selected-submodel null hypothesis $H_{0,j,\widehat{M}_s}:\beta _{j,\widehat{M}_s}=0$. For the full Cox model and the debiased Lasso, the corresponding null hypothesis is $H_{0,j}^F:\beta _j^F=0$ in the full model, with the debiased Lasso evaluated among variables selected by the preceding Lasso step. For the oracle model, the null hypothesis is defined in the oracle submodel containing the true active set. Thus, rejection decisions, power, and type I error are interpreted relative to the same method-specific target as the coverage calculations.

### Methods (M)

All investigated procedures follow a two-stage structure. In a first step, a subset of covariates is selected using either the standard or the adaptive Lasso. In a second step, a dedicated inference procedure is applied to construct post-selection confidence intervals (PSCIs) for the regression coefficients corresponding to the selected variables. Table 4 provides a structured overview of the considered methods, which differ in how they account for the variable selection step.

**Table 4:** Overview of methods investigated in this study

Method	Variable selection	Tuning	Inference
full	none	–	Wald CI
oracle	none	–	Wald CI
refit	lasso	$\lambda$-dependent	Wald CI after refitting the selected model
refit0	lasso	$\lambda$-dependent	Wald CI without refitting
split	lasso	$\lambda$-dependent	Wald CI based on sample splitting
debiased	lasso	$\lambda$-dependent	$PSCI^{DB}$out of Eq. (3)
exact psi	lasso	$\lambda$-dependent	Exact conditional PSCI

#### Baseline and naive comparators

To establish reference points for comparison, we included a full Cox model containing all available covariates as well as an oracle model restricted to the truly active coefficients.

In addition, we considered two naive post-selection refitting strategies that ignore selection-induced bias. The first refitting strategy is a standard refit approach, where an unpenalized Cox model is fitted to the variables selected by the Lasso and inference is based on conventional Wald confidence intervals obtained from the fully re-optimized partial likelihood. The second variant, denoted *refit0*, omits the additional Newton–Raphson update after variable selection and instead relies on the one-step estimator produced at the end of the penalized fitting procedure.

The inclusion of the refit0 variant is motivated by methodological considerations. Inspection of the implementation of exact post-selection inference for the Cox model in the selectiveInference package [37] reveals that the underlying estimator corresponds to such a one-step refit rather than a fully re-optimized partial likelihood fit. Including refit0 therefore allows us to disentangle the effect of the estimator itself from the additional conditioning step used in exact PSI and to assess how closely a naive one-step refit aligns with the estimators underlying theoretically justified post-selection inference procedures.

#### Post-selection inference methods

Beyond these baseline and naive approaches, we considered several methods that explicitly aim to provide valid inference after variable selection. These include sample splitting, which separates model selection and inference across independent data splits, the debiased Lasso, which applies a projection-based correction to mitigate shrinkage bias, and exact PSI, which conditions on the selection event to achieve conditional post-selection validity under the implemented selection event.

All Lasso-based procedures rely on a choice of the regularization parameter $\lambda$. The interaction between tuning strategy and post-selection inference validity is a central aspect of our empirical investigation.

#### Choice of tuning rules

The performance of both the standard and adaptive Lasso depends critically on the choice of the regularization parameter $\lambda$. To provide a comprehensive and practically relevant comparison, five tuning rules were considered, summarized in Table 5. These rules can be grouped into prediction-oriented, model-selection-oriented, and fixed approaches.

**Table 5:** Overview of tuning rules used to select the penalty parameter $\lambda$ in this study

Tuning rule	Tuning type	Description
min	CV-based	$\lambda$ minimizing cross-validated risk
1se	CV-based	Largest $\lambda$ within one standard error of the minimum CV risk
fix	fixed	Fixed, scenario-specific penalty parameter obtained from repeated Lasso fits on a large simulated population dataset
aic	information-based	$\lambda$ selected by Akaike Information Criterion
bic	information-based	$\lambda$ selected by Bayesian Information Criterion

First, two prediction-oriented tuning rules based on 10-fold cross-validation (CV) were used. The tuning parameter $\lambda _{\textrm{CV,min}}$ selects the value of $\lambda$ that minimizes the cross-validated partial likelihood risk, whereas the $\lambda _{\textrm{CV,1SE}}$ rule chooses the largest $\lambda$ whose cross-validated risk lies within one standard error of the minimum. The latter favors more frugal models while maintaining comparable predictive performance ([38], Section 7.10.1).

Second, two information-theoretic tuning rules were employed. The AIC-type choice $\lambda _{\textrm{AIC}}$ is defined by minimizing an Akaike-type information criterion and primarily targets predictive accuracy, whereas the BIC-type choice $\lambda _{\textrm{BIC}}$ imposes a stronger penalty on model complexity and tends to favor sparser models. Under suitable regularity conditions, BIC-type criteria are known to lead to consistent variable selection [39–41].

Finally, a fixed regularization parameter $\lambda _{\textrm{fix}}$ was included as a benchmark. This value was derived from a large-scale external dataset ($N=100{,}000$), where 1,000 repeated Lasso fits were performed to obtain a stable, scenario-specific estimate of an appropriate penalty level. The resulting value was kept fixed across Monte Carlo repetitions within each scenario and was intended to approximate a prespecified, near-oracle penalty choice. It is therefore the tuning regime most closely aligned with the fixed-$\lambda$ assumption underlying exact PSI. Technical details on the construction of this fixed tuning parameter and the definition of the AIC and BIC criteria are provided in Supplementary Material S1.2.

Taken together, this range of tuning rules reflects commonly used choices in applied Lasso analyses and allows for a systematic assessment of how different selection strategies interact with subsequent inference and variable selection properties across the methods listed in Table 4. Only $\lambda _{\textrm{fix}}$ is treated as prespecified. The CV-, AIC-, and BIC-based choices are data-adaptive and are included to reflect common applied workflows. For exact PSI, results under these data-adaptive tuning rules are therefore interpreted as pragmatic sensitivity analyses outside the strict fixed-$\lambda$ guarantee, unless the tuning procedure itself is included in the conditioning event.

### Performance measures (P)

Performance is evaluated using *post-selection coverage*, *PSCI width*, *post-selection power*, and *post-selection type I error*. All inferential performance measures are interpreted relative to the method-specific targets and coverage criteria described in Table 3. In addition, runtimes (in seconds) were recorded for each method, and model performance measures were reported.

Post-selection coverage is defined as the proportion of PSCIs that contain the relevant method-specific target coefficient among the selected variables and is assessed relative to the nominal 90% level. The reported coverage is an empirical post-selection coverage measure over repeated data-generating and selection steps. This is distinct from formal selection-conditional coverage guarantees, as provided by exact post-selection inference only under the conditioning event used by the method, for example with fixed Lasso tuning parameter $\lambda$ [4, 9, 13]. More generally, post-selection coverage depends on both the target estimand and how selection is accounted for [3, 23, 24].

For sample splitting, exact PSI, and refitting-based approaches, the reference target is the selected-submodel coefficient $\beta _{j,\widehat{M}}$. For data-adaptive tuning rules such as cross-validation, AIC, or BIC, exact PSI results are interpreted empirically rather than as finite-sample conditional validity guarantees. For the debiased Lasso, the reference target is the full-model coefficient $\beta _j^F$, with coverage evaluated only among variables selected by the preceding Lasso step [10, 11, 15]. Coverage rates closer to the nominal level indicate better calibration relative to the corresponding method-specific target and coverage criterion.

PSCI width corresponds to the average length of the post-selection confidence intervals and serves as a measure of estimation precision. Because the underlying targets differ across methods, interval widths are interpreted as practical reporting properties rather than as purely target-invariant efficiency comparisons.

Post-selection power quantifies the probability of rejecting the relevant method-specific null hypothesis when the corresponding target coefficient is non-zero. Post-selection type I error measures the probability of rejecting this null hypothesis when the corresponding target coefficient equals zero.

Detailed definitions and formulas for all primary performance measures are provided in Supplementary Material S1.3.

In addition to inferential performance, we evaluated predictive performance of the selected models as a secondary outcome. While the concordance index (C-index) is widely used to assess discrimination in survival analysis, it has been criticized for limited interpretability and sensitivity to censoring [42]. We therefore primarily assess predictive accuracy using the integrated Brier score (IBS), which aggregates time-dependent squared prediction errors over the follow-up period and captures both discrimination and calibration. The IBS was computed using inverse probability of censoring weights [43]. Lower values of the IBS indicate better predictive performance. C-index is reported for completeness and comparability with prior work.

To quantify the efficiency loss induced by variable selection, predictive performance was evaluated alongside model size and variable selection accuracy. An oracle model fitted with knowledge of the true active set serves as a theoretical benchmark. Corresponding oracle results are reported in supplementary analyses.

### Software and implementation details

All simulations and analyses were conducted in R (version 4.4.2). The survival package [25, 44] was used for fitting Cox models and handling time-to-event data. Penalized estimation via the Lasso and adaptive Lasso was carried out using the glmnet package [45–47].

Exact conditional post-selection inference was implemented via the selectiveInference package [37]. While this framework supports the Cox model under fixed penalty conditions, procedures for randomized or data-driven penalty selection are presently limited to the linear regression setting.

Methods without established, off-the-shelf software support for the Cox model were implemented manually based on their methodological descriptions in the literature. In particular, debiased inference for the Cox model was implemented following Lu and Xia [31], using their research code as a reference. All custom implementations and simulation code are provided in the supplementary material.

## Results

This section presents the results of the simulation study. We first report inferential performance for the post-selection intervals and associated hypothesis tests, interpreted relative to the method-specific targets defined in Table 3. We then summarize non-inferential performance measures related to prediction and variable selection.

### Post-selection inferential performance

Results for the primary inferential performance measures are reported in terms of post-selection coverage, PSCI width, post-selection power, and post-selection type I error. Consistent with the Methods (M) subsection, coverage, power, and type I error are interpreted relative to the method-specific target and coverage criterion, rather than as target-invariant method rankings. No strict numerical cutoffs are imposed, as the interpretation focuses on systematic patterns rather than isolated values.

#### Post-selection coverage

Post-selection coverage is evaluated as defined in the Methods (M) subsection and interpreted relative to the method-specific target and coverage criterion. Coverage probabilities closer to the nominal 90% level indicate better calibration for the target addressed by the respective method. Coverage is described as “high” when it remains close to the nominal 90% level with little variation across sample sizes, as “moderate” when noticeable but non-systematic deviations occur, and as “low” when substantial or persistent undercoverage is observed.

**Fig. 1:**
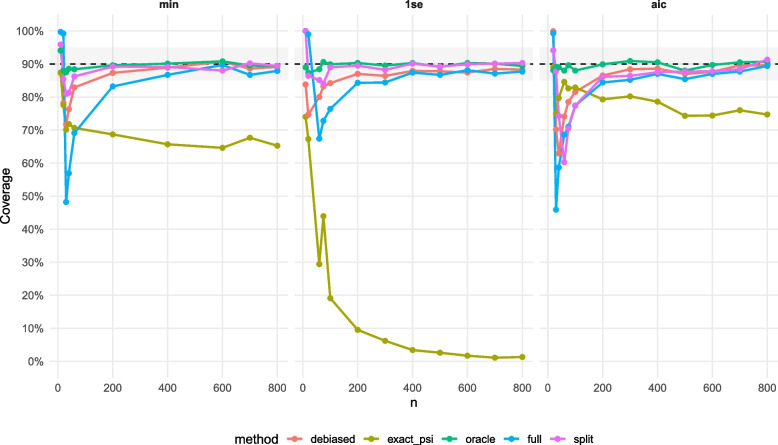
Post-selection coverage under the realistic coefficient pattern with $p=20$, Weibull baseline hazard, no censoring, and correlation $\rho =0.3$. Results are shown for coefficient $X_1$ using the non-adaptive Lasso with tuning choices $\lambda _{\textrm{CV,min}}$, $\lambda _{\textrm{CV,1se}}$, $\lambda _{\textrm{AIC}}$

Figure 1 illustrates post-selection coverage probabilities for a representative setting with a realistic coefficient pattern and $p=20$. For sample splitting and exact PSI, coverage is interpreted relative to the selected submodel coefficient. For the debiased Lasso, coverage is interpreted relative to the full-model coefficient among selected variables.

Sample splitting and the debiased Lasso show empirical coverage close to their respective target levels for moderate to large sample sizes ($n \ge 300$) in the representative no-censoring setting and in many supplementary scenarios. Because these procedures target different coefficients, this pattern is interpreted as method-specific calibration rather than a target-invariant ranking. For $\lambda _{\textrm{CV,1SE}}$, increased variability in model selection is associated with modest deviations from nominal coverage.

Exact PSI is most directly aligned with its formal assumptions under the fixed, scenario-specific $\lambda _{\textrm{fix}}$ setting. When exact PSI is combined with data-adaptive tuning, especially $\lambda _{\textrm{CV,min}}$ and $\lambda _{\textrm{CV,1SE}}$, pronounced undercoverage is observed. This behavior should not be interpreted as a failure of exact PSI under its intended fixed-$\lambda$ setting, but as a sensitivity analysis showing the practical consequences of applying the method outside its strict formal scope.

The apparent robustness of these patterns should be interpreted with some caution. The appendix contains additional sensitivity analyses over sample sizes, tuning strategies ($\lambda _{\textrm{fix}}$, $\lambda _{\textrm{AIC}}$, $\lambda _{\textrm{BIC}}$, $\lambda _{\textrm{CV,min}}$, $\lambda _{\textrm{CV,1SE}}$), and target censoring proportions. These analyses support the same qualitative ordering of the methods in the settings considered, but they also show that the magnitude of undercoverage, interval inflation, and power loss depends on censoring and tuning.

**Fig. 2:**
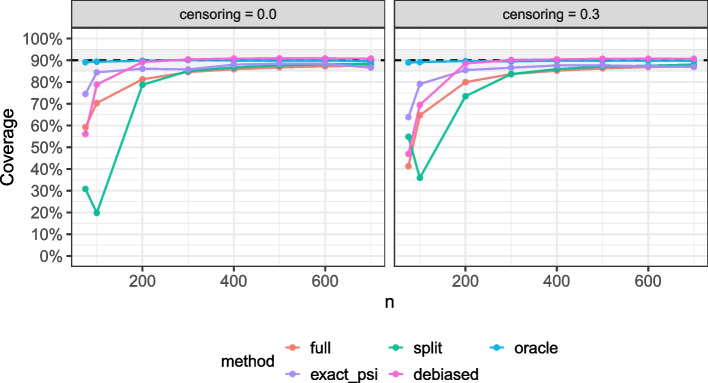
Post-selection coverage in a realistic toy setting with $p=50$, comparing no target censoring with 30% target censoring under the data-adaptive tuning rule $\lambda _{\textrm{AIC}}$. Results are shown across sample sizes and should be interpreted relative to the method-specific targets and coverage criteria

Figure 2 complements the no-censoring display in Fig. 1 by showing a direct censoring comparison in a more challenging realistic toy setting with $p=50$. The display uses the data-adaptive AIC tuning rule, for which finite-sample deviations are more visible than in some averaged displays. Increasing the target censoring proportion from 0 to 0.3 reduces the effective information and can make method-specific differences more apparent. These patterns are interpreted as empirical sensitivity analyses and not as target-invariant rankings, because the procedures address different method-specific targets and exact PSI is formally aligned with fixed-$\lambda$ selection.

**Fig. 3:**
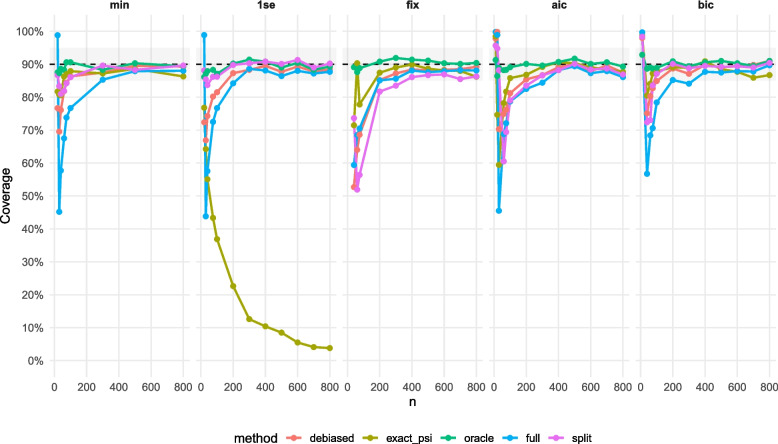
Post-selection coverage under the realistic coefficient pattern with $p=20$, Weibull baseline hazard, no censoring, and correlation $\rho =0.3$, using the adaptive Lasso for variable selection. Results are shown for coefficient $X_1$ across the tuning choices $\lambda _{\textrm{CV,min}}$, $\lambda _{\textrm{CV,1se}}$, $\lambda _{\textrm{fix}}$, $\lambda _{\textrm{AIC}}$, and $\lambda _{\textrm{BIC}}$

Figure 3 reports the corresponding no-censoring, $\rho =0.3$ coverage pattern when variable selection is performed with the adaptive Lasso. The adaptive Lasso changes the selected models through data-dependent penalty weights and may therefore affect sparsity, selection stability, and the variables for which PSCIs are reported. In the displayed scenario, the method-specific coverage patterns are qualitatively similar to the non-adaptive Lasso analysis, but this should be interpreted only as an empirical sensitivity analysis. In particular, adaptive penalization does not by itself provide a post-selection coverage guarantee, and the distinction between selected-submodel and full-model targets remains unchanged.

#### PSCI widths

PSCI width is used as a measure of inferential precision, with shorter intervals indicating greater practical precision for the reported interval. Because the methods do not always target the same coefficient, interval widths are interpreted together with the target definitions in Table 3.

**Fig. 4:**
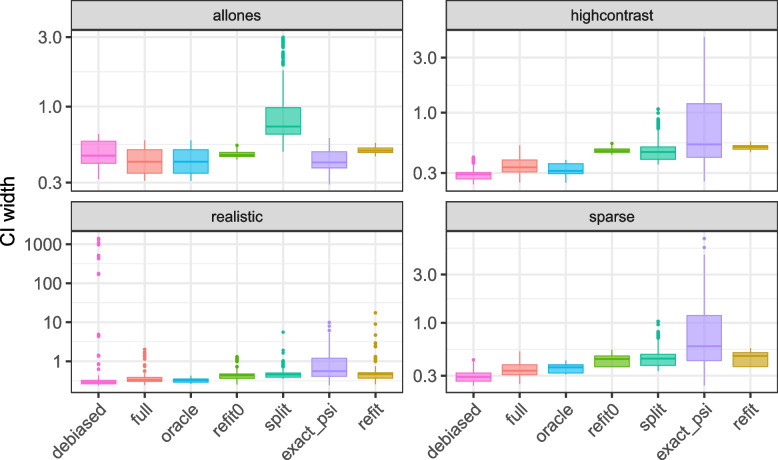
Distribution of PSCI lengths for selected coefficients at sample size $n=200$ across core inference methods in the toy simulation settings, shown on a log scale. Panels correspond to the four coefficient patterns

Figure 4 summarizes the distribution of PSCI lengths across inference methods for a representative toy setting. PSCI width provides a complementary perspective on post-selection inference performance by highlighting the practical consequences of different adjustment strategies.

Across scenarios, the debiased Lasso produces comparatively short and stable PSCIs for the full-model target among selected variables. Because this target differs from the selected-submodel target of sample splitting and exact PSI, the shorter intervals are interpreted as practical reporting properties rather than as target-invariant efficiency advantages. Sample splitting yields wider PSCIs with increased variability, particularly under the METABRIC-calibrated design, consistent with its reduced effective sample size.

Exact PSI produces the widest PSCIs overall, especially when combined with data-adaptive tuning. This reflects substantial uncertainty inflation due to conditioning on complex selection events and, for data-adaptive tuning rules, the practical mismatch between the implemented workflow and the fixed-$\lambda$ assumption. Increasing the sample size generally reduces PSCI widths for all methods, while the method-specific pattern of practical interval lengths is largely preserved in the settings considered.

#### Post-selection power and type I error

Post-selection power and post-selection type I error are evaluated as defined in Methods (M) section and quantify the ability to detect non-zero method-specific target effects while controlling false rejections after variable selection.

Overall, procedures designed for post-selection inference provide a more appropriate basis for interpreting power and type I error than naive refitting, but the operating characteristics remain target-specific. In particular, the debiased Lasso maintains type I error rates close to the nominal level while retaining high power for the full-model target among selected variables, whereas sample splitting exhibits inflated type I error rates despite reasonable power in several scenarios. In contrast, naive refitting and procedures not designed for post-selection inference tend to be liberal.

**Fig. 5:**
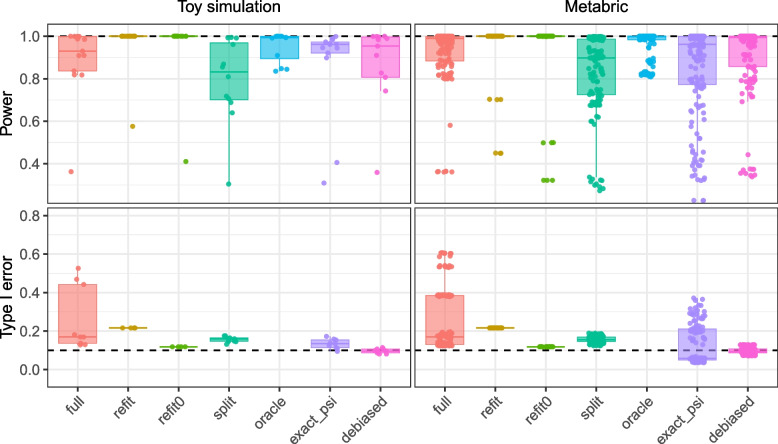
Post-selection power (top row) and type I error rates (bottom row) for the toy and METABRIC settings at sample size $n=75$. The dashed horizontal line indicates the nominal type I error level

Figure 5 reports post-selection power and post-selection type I error rates averaged over multiple simulation scenarios for the toy and METABRIC designs at sample size $n=75$. Averaging across scenarios reduces setting-specific variability and highlights systematic differences between inference procedures.

Results for larger sample sizes ($n=100$ and $n=200$), alternative tuning rules ($\lambda _{\textrm{CV,min}}$, $\lambda _{\textrm{CV,1se}}$, $\lambda _{fix}$, $\lambda _{\textrm{AIC}}$, $\lambda _{\textrm{BIC}}$), and different target censoring proportions are reported in Supplementary Material S2.3. Rather than claiming invariance across all designs, we use these additional analyses to identify which features are stable and which are censoring-sensitive.

**Fig. 6:**
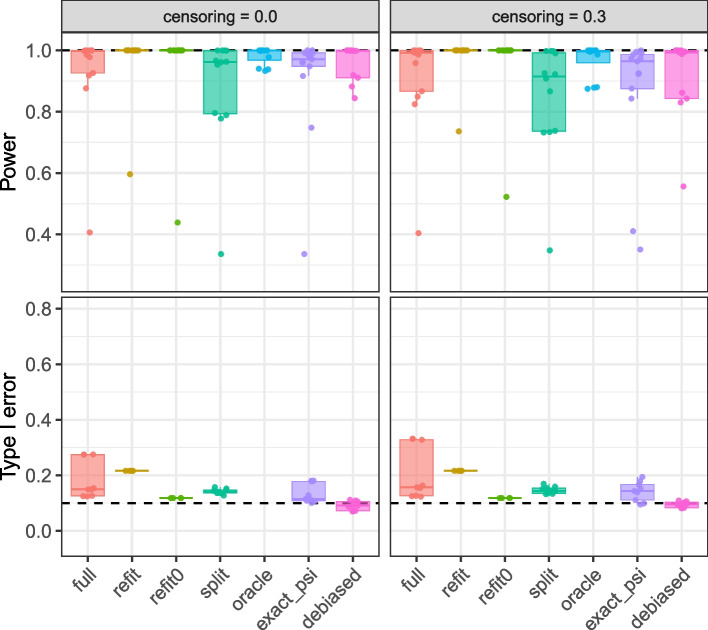
Post-selection power and post-selection type I error stratified by target censoring proportion for the toy setting with $\lambda _{\textrm{CV,min}}$ and $n=100$. The figure complements the averaged results in Fig. 5 by showing the direct effect of 30% target censoring

Increasing the sample size improves post-selection power and reduces variability in type I error rates in most settings. Less regularized tuning strategies, such as $\lambda _{\textrm{CV,min}}$ or $\lambda _{\textrm{fix}}$ rules, yield higher post-selection power but exhibit greater variability in type I error control, particularly under the METABRIC design. More conservative choices improve stability at the cost of reduced power. Figure 6 isolates the role of censoring for $\lambda _{\textrm{CV,min}}$ at $n=100$. The main change induced by 30% target censoring is a reduction in post-selection power, with some additional variability in type I error; the method-specific pattern remains broadly similar, but the separation between procedures becomes more visible.

### Performance measures

Predictive performance of the selected models was evaluated as a secondary outcome. Results are summarized using the integrated Brier score (IBS), with lower values indicating better predictive accuracy. Selection-related metrics such as average model size and the proportion of truly active variables among the selected set ($P_{\text {true}}$) are reported alongside IBS. Corresponding results for the concordance index (C-index) are provided in Supplementary Material S2.5.

Across simulation settings, prediction-oriented tuning rules, in particular the AIC-type criterion and $\lambda _{\textrm{CV,min}}$, most often achieved the lowest IBS. However, these rules selected substantially larger models and yielded markedly lower $P_{\text {true}}$, especially for $p=50$.

More parsimonious tuning rules, such as $\lambda _{\textrm{CV,1SE}}$ and the BIC-type criterion $\lambda _{\textrm{BIC}}$, selected considerably smaller models and achieved higher values of $P_{\text {true}}$, while incurring only a modest loss in predictive performance. Overall, these results illustrate the expected trade-off between prediction accuracy and sparsity: tuning strategies optimized for prediction tend to favor dense models, whereas selection-oriented rules yield more interpretable solutions with improved selection quality.

### Summary of main results

Across the simulated settings considered, procedures designed for post-selection inference provide substantially improved method-specific inferential calibration compared to naive post-selection approaches. These comparisons must be interpreted relative to the targets and coverage criteria defined in Table 3: sample splitting and exact PSI are selected-submodel oriented, whereas the debiased Lasso targets full-model coefficients and is evaluated among selected variables.

The debiased Lasso and sample splitting show empirical coverage close to their respective nominal targets in many of the examined settings, although their formal targets differ and performance is less stable in smaller samples or under heavier censoring. Exact PSI is sensitive to the choice of the regularization parameter. Its fixed-$\lambda$ results are closest to the assumptions underlying the method, whereas data-adaptive tuning rules such as cross-validation are best understood as pragmatic sensitivity analyses outside the strict formal guarantee.

The adaptive Lasso results included in the main manuscript do not materially change the displayed method-specific patterns. Adaptive penalization can change model sparsity, selection stability, and hence the set of coefficients for which intervals are reported. However, it does not remove the need to distinguish inferential targets or to account for data-adaptive tuning. Consequently, the adaptive Lasso figures are best read as additional sensitivity analyses rather than as separate validity guarantees.

In terms of practical interval reporting, the debiased Lasso consistently yields shorter PSCIs than sample splitting and exact PSI for its full-model target among selected variables. Exact PSI can incur substantial interval inflation due to conditioning on complex selection events, while sample splitting is affected by the reduced effective sample size of the inference split. Differences between methods diminish with increasing sample size but remain visible in moderate-dimensional settings.

Accordingly, type I error is described as “controlled” when empirical rates remain close to the nominal level, as “inflated” when systematic over-rejection is observed, and as “conservative” when rejection rates are consistently below the nominal level. Post-selection power and post-selection type I error exhibit clear and method-specific trade-offs, which are summarized qualitatively in Table 6. In this summary, power is described as high or moderate relative to the oracle benchmark, while type I error behavior is classified as controlled, inflated, or conservative based on systematic deviations from the nominal level for the corresponding method-specific null hypothesis.

**Table 6:** Qualitative summary of method-specific post-selection inferential performance across methods and tuning strategies

Method	Tuning category	Coverage	Power	Type I error
split	fixed	often close to nominal for the selected-submodel target; more variable in small samples	moderate	sometimes inflated
split	CV	often close to nominal for the selected-submodel target; censoring-sensitive	moderate	sometimes inflated
debiased	fixed	often close to nominal for the full-model target among selected variables	comparatively high	mostly controlled
debiased	CV	often close to nominal for the full-model target among selected variables; target-specific interpretation required	comparatively high	mostly controlled
exact PSI	fixed	most closely aligned with its formal fixed-$\lambda$validity setting; finite-sample variability remains	low to moderate	conservative
exact PSI	CV/data-adaptive	often below nominal when used outside the fixed-$\lambda$guarantee	low	conservative

Predictive performance, as measured by the IBS, is comparatively insensitive to the choice of inference procedure and tuning strategy. Across methods, predictive accuracy remains relatively stable, even when inferential properties differ substantially, and efficiency losses relative to the oracle model are moderate. Overall, the results highlight a clear distinction between inferential validity and predictive performance in Cox models after variable selection.

## Real data example

We illustrate the proposed post-selection inference framework using data from the Molecular Taxonomy of Breast Cancer International Consortium (METABRIC) study [35], a large breast cancer cohort with long-term clinical follow-up. Clinical data were obtained from cBioPortal. Only publicly available clinical variables were used to ensure full reproducibility of the analysis. No controlled-access molecular or raw genomic data were accessed.

### Research question

The METABRIC data example serves as an illustration of variable selection and post-selection inference in a clinically relevant right-censored survival setting. The focus of this analysis is methodological rather than etiological: the goal is not causal interpretation or biomarker discovery, but prognostic modeling based on routinely available clinical covariates.

Specifically, we aim to investigate: (i) the stability of variable selection across different Lasso-based methods and tuning strategies, (ii) the resulting post-selection confidence intervals (PSCIs) for selected covariates, and (iii) how different post-selection inference approaches quantify uncertainty in a data-adaptive modeling workflow.

Overall survival time (in months) was defined as overall_survival_months, and the event indicator was derived from patients_vital_status (death vs. censoring). We considered routinely available clinical predictors, including age at diagnosis, tumor stage, ER status, HER2 status, PR status, chemotherapy, hormone therapy, radiotherapy, histologic grade, tumor size, number of positive lymph nodes, and the Nottingham Prognostic Index (NPI).

### Analysis

The METABRIC dataset was analyzed using the same inferential procedures as in the simulation study, including the full Cox model, sample splitting, the debiased Lasso, and exact PSI.

To assess the stability of variable selection, we followed an approach similar to Kammer et al. [16]. Specifically, we performed 100 subsampling repetitions. In each repetition, the variable selection step was re-run, and selection frequencies were computed as the proportion of subsamples in which each covariate was selected.

In parallel, confidence intervals were recomputed for each subsample and inference method. This allows a joint assessment of selection stability and uncertainty quantification across methods, rather than relying on a single realization of the selected model.

Unless stated otherwise, results are reported for the non-adaptive Lasso with the cross-validated tuning choice $\lambda _{\textrm{CV,min}}$, which reflects a commonly used applied workflow. Alternative tuning rules ($\lambda _{\textrm{AIC}}$, $\lambda _{\textrm{CV,1SE}}$) yield qualitatively similar patterns and are reported in the Supplementary Material.

### Results

**Fig. 7:**
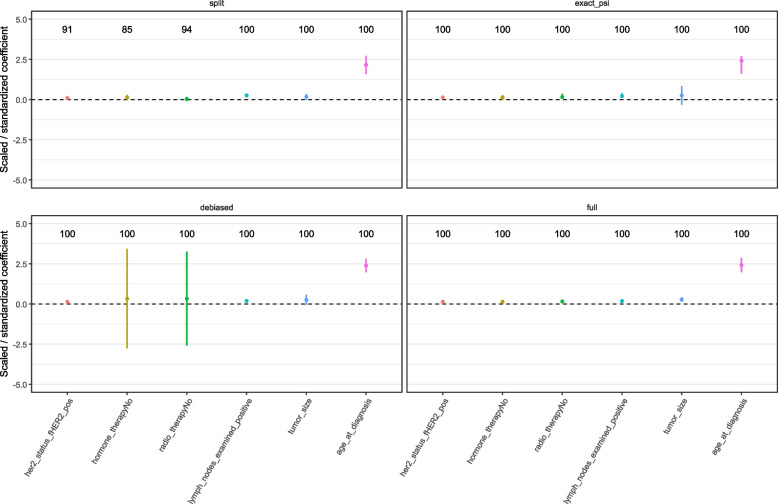
Real data example METABRIC: point estimates and 90% post-selection confidence intervals for regression coefficients obtained with different inference methods. Results are shown for the cross-validated tuning choice $\lambda _{\textrm{CV,min}}$. Coefficients are displayed on the original scale and ordered by increasing standardized effect size. Numbers above the panels indicate selection frequencies (in %) across 100 subsamples

Figure 7 summarizes the estimated regression coefficients and corresponding 90% post-selection confidence intervals for the METABRIC data. The presentation follows the layout introduced by Kammer et al. [16]. Results are shown for the cross-validated tuning choice $\lambda _{\textrm{CV,min}}$; the corresponding figures for $\lambda _{\textrm{CV,1SE}}$ and the AIC-based tuning rule were qualitatively very similar and are therefore omitted for brevity.

Across inference methods, point estimates are broadly comparable, whereas the width and stability of the post-selection confidence intervals differ substantially. Sample splitting and exact PSI tend to produce wider intervals overall, reflecting the explicit adjustment for the variable selection step. In contrast, the debiased Lasso often yields comparatively narrower intervals, albeit with increased variability across subsamples.

A more detailed inspection reveals that these patterns depend strongly on the covariate type. For continuous predictors, the debiased Lasso generally provides more precise post-selection inference, resulting in relatively narrow confidence intervals. However, for binary or ordinal covariates, the debiased Lasso exhibits pronounced interval inflation, leading to substantially wider confidence intervals. Exact PSI shows the opposite behavior: while it produces wider intervals for continuous covariates than the debiased Lasso, it yields more stable and interpretable post-selection confidence intervals for binary and ordinal predictors.

Selection frequencies further support this distinction. Core clinical predictors such as tumor size and tumor stage are selected consistently across subsamples and inference procedures. In contrast, treatment-related covariates (e.g., chemotherapy or radiotherapy) exhibit considerable method-dependent variability, reflecting both correlation structures in the data and differences in how selection uncertainty is propagated into inference.

Overall, the METABRIC example illustrates how post-selection inference can be used to distinguish between stable and unstable prognostic signals in a realistic clinical setting. Rather than interpreting individual confidence intervals in isolation, the joint consideration of selection frequencies and post-selection confidence intervals provides a transparent summary of both variable importance and inferential uncertainty in a data-adaptive modeling pipeline.

## Discussion

In this work, we conducted a neutral comparison study of inference procedures applied after Lasso-type variable selection in Cox proportional hazards models. Our objective was not to identify a universally optimal method, but to clarify the inferential guarantees, efficiency trade-offs, and practical sensitivities that arise when data-driven variable selection and tuning are combined with post-selection uncertainty quantification in survival analysis.

A central implication of our results is that uncertainty quantification after variable selection depends critically on aligning the inferential target with the intended analysis goal. In many applied settings, particularly exploratory or prognostic modeling, the primary interest lies in the coefficients of the selected submodel, rather than in recovering a single underlying “true” full model. From this submodel-oriented perspective, procedures that explicitly account for the selection step are appealing because they address the optimism introduced by variable screening and support more reproducible reporting of uncertainty. At the same time, the debiased Lasso targets a different estimand, namely a full-model coefficient, and should therefore be interpreted from a different inferential viewpoint.

Taken together, the simulations illustrate that methods designed for post-selection inference can improve method-specific post-selection calibration relative to naive post-selection refitting in the scenarios considered. However, these improvements depend on the target estimand, the tuning strategy, the sample size, and the amount of censoring. This reinforces the need to interpret coverage, power, and type I error relative to the method-specific target and null hypothesis, rather than as target-invariant performance measures. For exact PSI, this distinction is particularly important because its formal post-selection guarantee is tied to a fixed selection event. Accordingly, results for exact PSI under cross-validation, AIC, or BIC should be read as pragmatic evaluations of common applied workflows rather than as settings covered by the formal fixed-$\lambda$ guarantee.

Our results also highlight that selection adjustment is not cost-free. Methods that condition strongly on the selection event tend to produce wider post-selection confidence intervals, reflecting uncertainty inflation after variable selection. Sample splitting provides a conceptually simple and transparent alternative, but uses only part of the data for inference and may therefore lose precision, particularly when the effective number of events is limited by censoring. The debiased Lasso often provides shorter intervals, but these intervals refer to the full-model target among selected variables and therefore answer a different inferential question. Thus, differences in interval width should be interpreted as practical reporting properties under different inferential targets, not as purely target-invariant efficiency comparisons.

The choice of tuning strategy for the regularization parameter $\lambda$ emerged as an important practical factor. Less conservative tuning rules tend to favor larger selected models, whereas more conservative rules promote sparsity. This creates a trade-off between selection stability, power, interval width, and error control, and this trade-off cannot be separated from the inferential target of the method. These differences were more pronounced for inferential performance than for predictive performance, suggesting that tuning choices that appear similar from a prediction perspective may still lead to different post-selection inferential behavior.

Censoring further complicates this trade-off. Increasing the censoring proportion reduces the effective information available for both variable selection and inference, and may therefore affect post-selection power and calibration. This is particularly relevant for procedures that rely on strong conditioning, reduced inference samples, or approximate variance estimation. Our findings therefore support the need to assess post-selection inference procedures under censoring regimes that are realistic for biomedical survival studies, rather than only under idealized low-censoring settings. At the same time, the present simulations do not exhaustively cover all combinations of high censoring, stronger covariate correlation, and sparse signal structures. The observed censoring-related differences should therefore be interpreted as design-dependent sensitivity patterns, not as a complete robustness assessment.

The real-data analysis based on the METABRIC cohort illustrates these methodological considerations in a clinically relevant setting. Rather than focusing on causal interpretation, the analysis highlights how post-selection inference can be used to distinguish stable prognostic signals from weaker or unstable associations. Combining selection frequencies with post-selection confidence intervals provides a transparent summary of both variable importance and uncertainty, and avoids overconfident interpretation of effects that arise from data-adaptive modeling pipelines. In this context, differences between inference methods are best understood as reflecting different use cases: conservative approaches may be preferable when controlling false discoveries is paramount, whereas more efficient procedures may be attractive in exploratory settings where power and interval width are critical.

Our study has several limitations. First, the simulation designs focus on a finite set of coefficient patterns and baseline hazard specifications. While we varied sample size, censoring, correlation structure, and tuning strategies, not all combinations of high censoring and stronger covariate correlation were exhaustively examined, and the censoring-stratified analyses should therefore be interpreted as sensitivity analyses rather than as complete robustness checks. More complex features such as time-varying effects, interactions, or non-proportional hazards were not considered. Second, the investigated methods do not share a single inferential target. Sample splitting and exact PSI are naturally interpreted relative to selected-submodel coefficients, whereas the debiased Lasso targets full-model coefficients. Although this reflects how these methods are commonly used after variable selection, it limits purely target-invariant comparisons of coverage and interval width. Alternative estimands, such as target-population parameters under model misspecification, may lead to different conclusions regarding the relative merits of the methods.

Third, implementation details matter. Default software choices were necessary for comparability, but applied analyses may benefit from problem-specific calibration of variance estimation, numerical tolerances, or tuning strategies. In particular, the implemented exact PSI procedure conditions on the selected model for a fixed penalty parameter, but does not condition on data-adaptive tuning steps such as cross-validation, AIC, or BIC.

Fourth, the present implementation of sample splitting uses a single 50/50 split. Although this choice provides a transparent baseline, it does not address the additional Monte Carlo variability induced by the random split. Multiple splitting provides a principled way to reduce the sensitivity to an arbitrary split by repeating the selection–inference procedure and aggregating evidence across repetitions [30]. Extensions based on e-values are methodologically attractive because e-values can be aggregated in several settings and can support selected-inference guarantees under suitable conditions [48, 49]. However, such approaches would require Cox-specific e-value-based confidence intervals under right censoring and are therefore best viewed as future methodological work rather than as directly available comparators in the present study.

Despite these limitations, the consistency of qualitative conclusions across a wide range of scenarios provides reassurance that the main insights generalize to realistic analysis workflows. Overall, our results emphasize that carefully targeted post-selection inference can substantially improve the credibility of post-selection conclusions in Cox models, but should be viewed as part of a broader modeling strategy that includes careful tuning, sensitivity analyses, and explicit communication of the intended inferential target.

## Supplementary Information

Supplementary Material 1.

## Data Availability

All data analyzed in this study are publicly available from cBioPortal: https://www.cbioportal.org/study/summary?id=brca_metabric.
