# Supplementary material for: Statistical inference after variable selection in Cox models: a neutral simulation study
Source: BMC Med Res Methodol. 2026 Jun 23;26:143. doi: 10.1186/s12874-026-02887-0 (PMC13289528; doi:10.1186/s12874-026-02887-0)
Supplement: Supplementary file 1 — Supplementary Material 1. [file 12874_2026_2887_MOESM1_ESM.pdf]

Supplementary Material for: Statistical inference after variable selection in Cox models: A simulation study

Lena Schemet[1] and Sarah Friedrich-Welz[1,2]

[1]Mathematical Statistics and Artificial Intelligence in Medicine, University of Augsburg, Augsburg, 86159, Bavaria, Germany.
[2]Center for Advanced Analytics and Predictive Sciences (CAAPS), University of Augsburg, Augsburg, 86159, Bavaria, Germany.

S1 Additional information - simulation study design

S1.1 Realistic simulation design

S1.1.1 Choice of the parametric survival model.

For the realistic simulation setting, several parametric proportional hazards models were considered for generating event times, including exponential, Weibull, log-normal, and log-logistic models using the `flexsurv` package [**?**]. Model choice was guided by empirical discrimination performance, assessed via time-dependent area under the ROC curve (AUC) in preliminary simulation runs. The Weibull model consistently achieved the highest AUC across a range of sample sizes and censoring levels, and was therefore selected as the data-generating mechanism for the realistic simulation scenarios reported in the main text.

S1.2 Additional methodological details

S1.2.1 Definition of AIC- and BIC-type tuning rules

For Lasso-based variable selection, the regularization parameter λ was selected by minimizing information criteria of the form

$$\mathrm{IC}(\lambda) = -2\,\ell(\hat{\beta}_\lambda) + c_n\,\mathrm{df}(\lambda),$$

where $\ell(\hat{\beta}_\lambda)$ denotes the partial log-likelihood of the Cox model evaluated at the Lasso estimate for a given λ, and $\mathrm{df}(\lambda)$ represents the effective degrees of freedom, defined as the number of nonzero coefficients selected by the Lasso [38].

For the AIC-type criterion, the penalty constant was set to $c_n = 2$, favoring models with good predictive performance [39]. For the BIC-type criterion, the penalty constant was chosen as $c_n = \log(n)$, imposing a stronger penalty on model complexity and encouraging more parsimonious models [40]. The selected tuning parameters were given by

$$\lambda_{\mathrm{AIC}} = \arg\min_{\lambda} \mathrm{AIC}(\lambda), \qquad \lambda_{\mathrm{BIC}} = \arg\min_{\lambda} \mathrm{BIC}(\lambda).$$

These criteria are commonly used in penalized likelihood frameworks and are known to exhibit different asymptotic properties with respect to prediction accuracy and variable selection consistency. In particular, AIC-type criteria are typically associated with good predictive performance, whereas BIC-type criteria impose a stronger complexity penalty and can lead to consistent model selection under suitable regularity conditions [38–41].

S1.2.2 Fixed regularization parameter

This section provides additional methodological details on the determination of the fixed regularization parameter used in the simulation study.

To obtain a scenario-specific fixed regularization parameter λ_{fix}, a preliminary large-sample study was conducted. For selected simulation scenarios, datasets of size ranging from 10^5 to 10^6 observations were generated. For each dataset, 1,000 independent Lasso fits using 10-fold cross-validation were performed, yielding corresponding values of $\lambda_{\mathrm{CV,min}}$.

The empirical distributions of $\lambda_{\mathrm{CV,min}}$ were summarized by their mean and median, together with associated standard errors. As the sample size increased, both summary measures exhibited a clear plateau behavior, with only negligible changes observed for sample sizes exceeding 100,000 observations (Figure S1).

Based on this stability, the procedure was subsequently standardized by generating a single dataset of size $n = 100{,}000$ for each simulation scenario. For each such dataset, 1,000 repeated Lasso fits with 10-fold cross-validation were performed, and the resulting mean value of $\lambda_{\min}$ was used to define the scenario-specific fixed regularization parameter λ_{fix}. These fixed values were then employed in the main simulation study to assess the impact of a stable, externally determined penalty parameter on variable selection and post-selection inference.

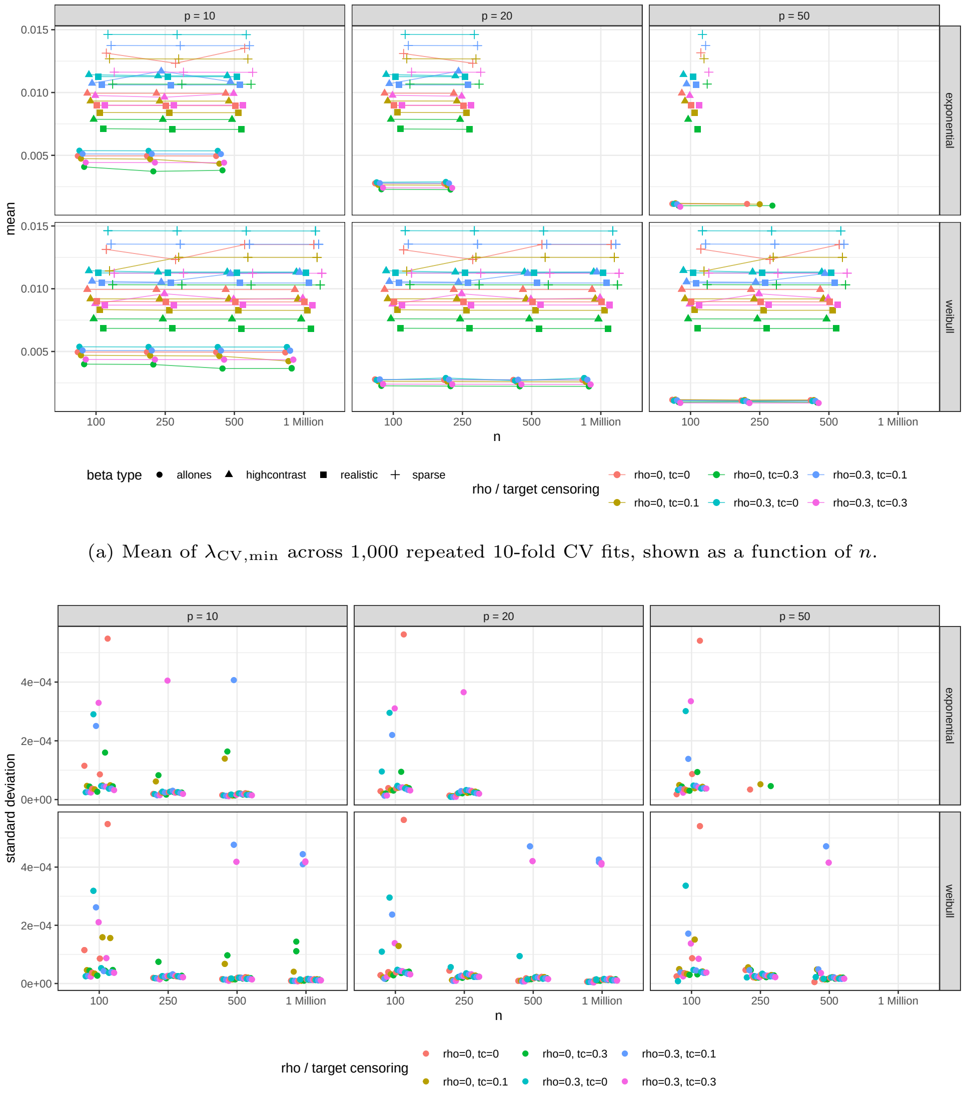

(a) Mean of $\lambda_{\text{CV,min}}$ across 1,000 repeated 10-fold CV fits, shown as a function of n.

(b) Standard deviation of $\lambda_{\text{CV,min}}$ across 1,000 repeated 10-fold CV fits, shown as a function of n.

Fig. S1: Stability of the cross-validated tuning parameter $\lambda_{\text{CV,min}}$ in the large-sample pre-study. Across scenarios, both the mean and variability of $\lambda_{\text{CV,min}}$ exhibit a plateau for large sample sizes, motivating the use of $n = 100{,}000$ to define scenario-specific fixed values λ_{fix}.

S1.3 Performance measures: formal definitions and notation

The performance measures used in this simulation study follow the framework introduced by Kammer et al. [16]. A detailed theoretical justification is provided therein.

Let $S = \{1, \ldots, n_{\text{sim}}\}$ denote the set of simulation iterations. For iteration $s \in S$, let $\widehat{M}_s \subseteq M_F$ denote the model selected by a given method, where M_F denotes the full set of predictors. For a selected model M, let $CI_{j,M}$ denote the post-selection confidence interval (PSCI) for coefficient β_j.

We denote the true regression coefficient by β_j^0 and use $\mathbf{1}(\cdot)$ for the indicator function. In the analyses reported in the main manuscript, the coefficient in these definitions is interpreted as the method-specific target: the selected-submodel coefficient for sample splitting, exact PSI, and refitting-based approaches, and the full-model coefficient for the debiased Lasso.

S1.3.1 Post-selection coverage

Post-selection coverage is evaluated empirically over repeated data-generating and selection steps as the proportion of PSCIs that contain the relevant method-specific target coefficient among selected variables. This empirical measure is distinct from a formal selection-conditional coverage guarantee for a fixed selection event. Based on the formula of Kammer et al. [16], we calculate/approximate post-selection coverage with the following formula:

$$\mathbb{P}\left[\beta_j^0 \in CI_{j,\widehat{M}} \,\middle|\, \widehat{M} = M\right] = \frac{\sum_{j \in M_F} \sum_{M \subseteq M_F} \sum_{s \in S} \mathbf{1}\left[\widehat{M}_s = M \;\wedge\; \beta_j^0 \in CI_{j,M}\right]}{\sum_{j \in M_F} \sum_{s \in S} \mathbf{1}[j \in \widehat{M}_s]}.$$

The numerator counts the number of simulation replicates where the CI contains the relevant target coefficient. The denominator counts the number of times coefficient j was actually selected.

S1.3.2 Post-selection power

Post-selection power measures the probability that a PSCI excludes zero when the corresponding method-specific target coefficient is non-zero:

$$\mathbb{P}\left[0 \notin CI_{j,\widehat{M}} \,\middle|\, \beta_j^0 \neq 0,\; \widehat{M} = M\right] = \frac{\sum_{j \in M_F} \sum_{M \subseteq M_F} \sum_{s \in S} \mathbf{1}\left[\widehat{M}_s = M \;\wedge\; 0 \notin CI_{j,M}\right] \mathbf{1}[\beta_j^0 \neq 0]}{\sum_{j \in M_F} \sum_{s \in S} \mathbf{1}[j \in \widehat{M}_s \;\wedge\; \beta_j^0 \neq 0]}.$$

Thus, power is evaluated only for coefficients that are truly non-zero and selected into the model.

S1.3.3 Post-selection type I error

Post-selection type I error quantifies the probability of falsely excluding zero from a PSCI when the corresponding method-specific target coefficient equals zero:

$$\mathbb{P}\Big[0 \notin CI_{j,\widehat{M}} \;\Big|\; \beta_j^0 = 0,\; \widehat{M} = M\Big] = \frac{\sum_{j \in M_F} \sum_{M \subseteq M_F} \sum_{s \in S} \mathbf{1}\Big[\widehat{M}_s = M \;\wedge\; 0 \notin CI_{j,M}\Big] \; \mathbf{1}[\beta_j^0 = 0]}{\sum_{j \in M_F} \sum_{s \in S} \mathbf{1}[j \in \widehat{M}_s \;\wedge\; \beta_j^0 = 0]}.$$

This measures the probability of a false rejection, conditional on both true null and selection.

S2 Additional results simulation study

This appendix section provides additional simulation results that complement the main findings reported in Section 5. The figures summarize post-selection coverage, confidence interval length, post-selection power, type I error rates, and computational runtimes across a range of simulation scenarios.

S2.1 Post-selection coverage

This subsection reports post-selection coverage probabilities for sample-splitting–based confidence intervals under various data-generating mechanisms, target censoring proportions, and tuning strategies.

S2.1.1 Toy simulation settings

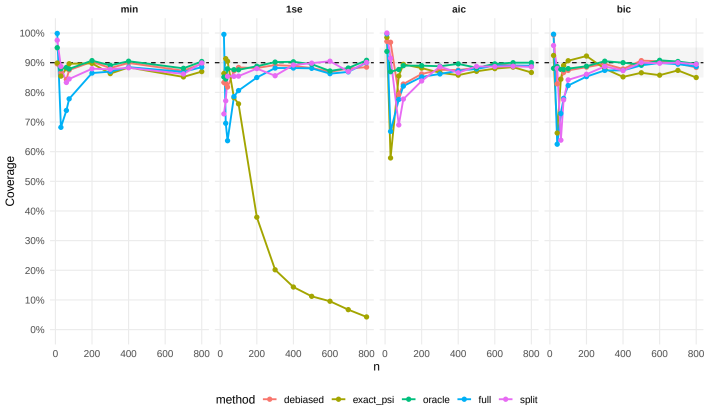

(a) Target censoring proportion 0.3, $\rho = 0.1$ (adaptive Lasso; $\lambda_{\mathrm{CV,min}}/\lambda_{\mathrm{CV,1se}}/\lambda_{\mathrm{AIC}}/\lambda_{\mathrm{BIC}}$).

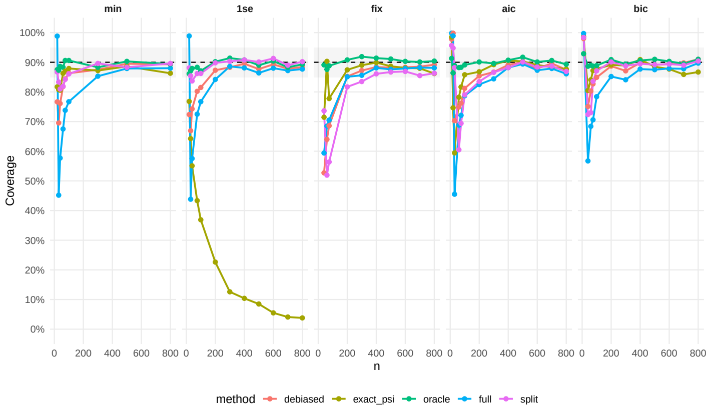

(b) Target censoring proportion 0.0, $\rho = 0.3$ (adaptive Lasso; $\lambda_{\mathrm{CV,min}}/\lambda_{\mathrm{CV,1se}}/\lambda_{\mathrm{AIC}}/\lambda_{\mathrm{BIC}}$).

Fig. S2: Post-selection coverage probabilities under the high-contrast coefficient pattern ($p = 20$; Weibull baseline) for the sample-splitting approach. Panels compare (a) no censoring and (b) 30% target censoring. Coverage is reported for coefficient X_1.

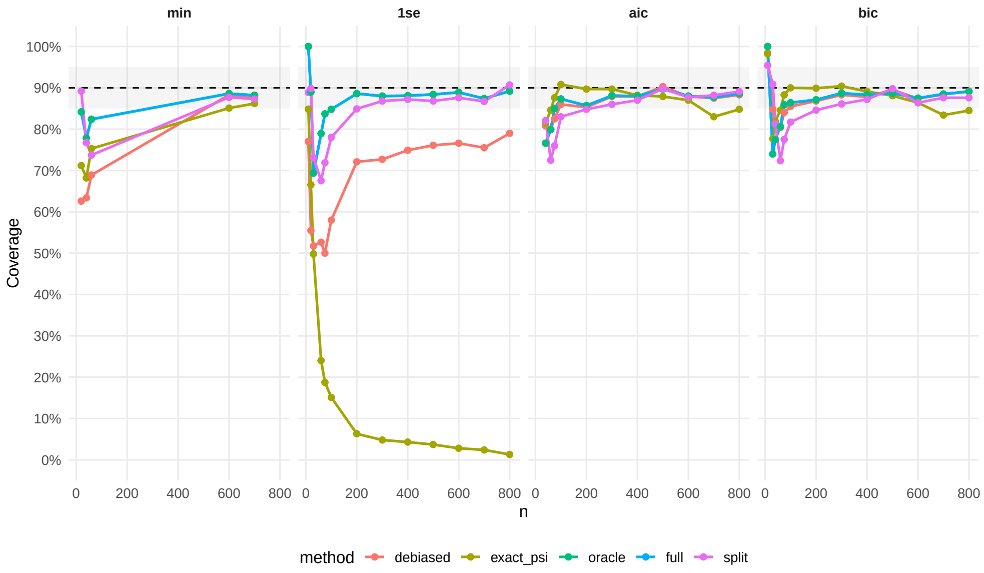

(a) Coefficient X_1 (non-adaptive Lasso; $\lambda_{\text{CV,min}}/\lambda_{\text{CV,1se}}/\lambda_{\text{AIC}}/\lambda_{\text{BIC}}$; target censoring $=$ 0.3, $\rho = 0.1$).

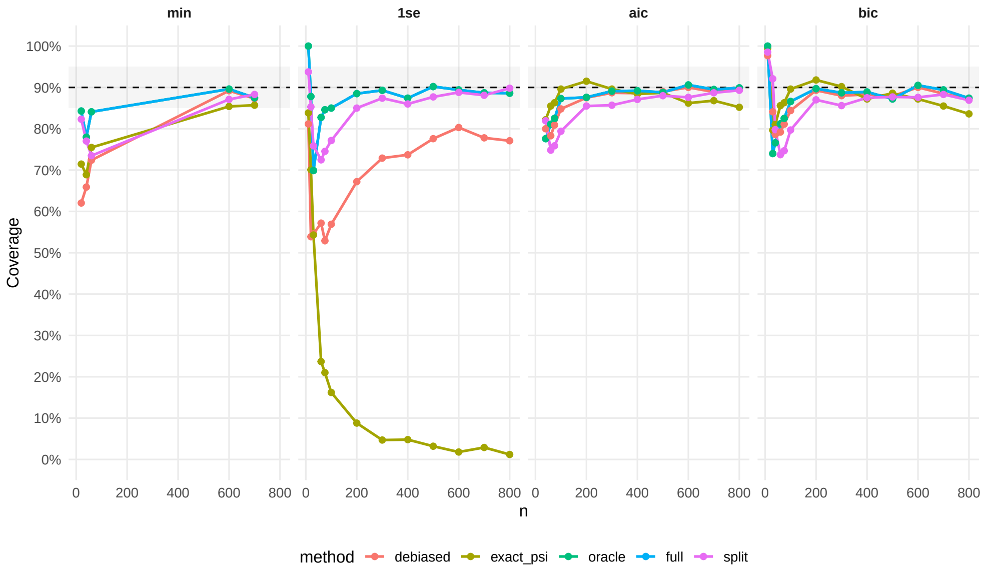

(b) Coefficient X_3 (non-adaptive Lasso; $\lambda_{\text{CV,min}}/\lambda_{\text{CV,1se}}/\lambda_{\text{AIC}}$; target censoring $= 0.3$, $\rho =$ 0.1).

Fig. S3: Post-selection coverage probabilities under the all-ones coefficient pattern ($p = 10$; Weibull distribution) for toy simulation. Panels show coverage for (a) coefficient X_1 and (b) coefficient X_3 under the same censoring and correlation setting (target censoring $= 0.3$, $\rho = 0.1$).

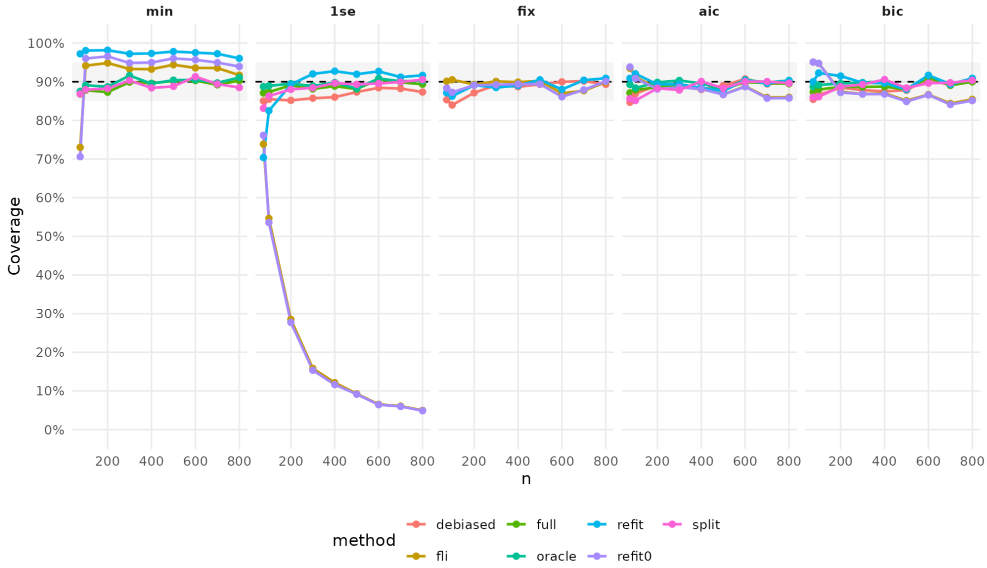

Fig. S4: Post-selection coverage under the high-contrast coefficient pattern with $p = 10$, a Weibull baseline distribution, no censoring ($\text{tc} = 0$), and independent covariates ($\rho = 0$). Results are shown for coefficient X_3 as a function of the sample size n across tuning choices.

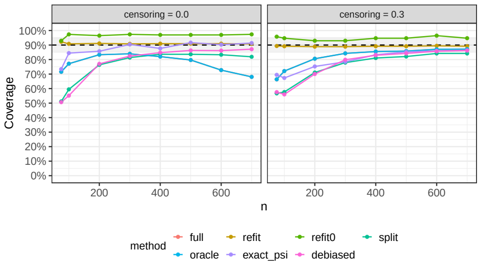

(a) All-ones pattern.

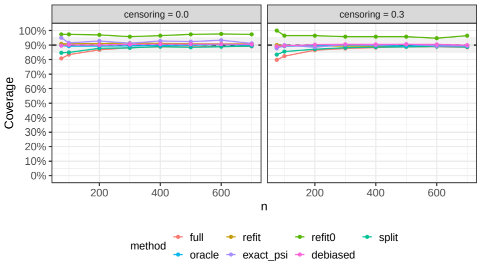

(b) High-contrast pattern.

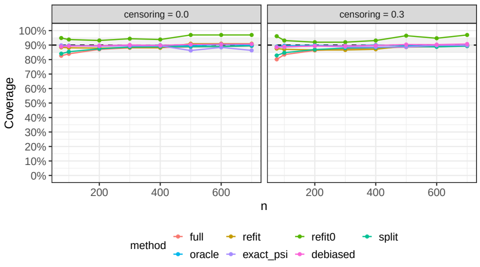

(c) Realistic pattern.

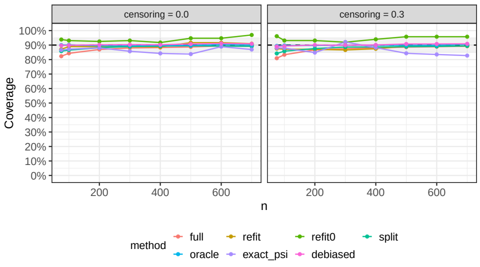

(d) Sparse pattern.

Fig. S5: Post-selection coverage in the toy simulations (simple setting) for the tuning choice $\lambda_{\mathrm{CV,min}}$ across sample sizes (n = 75–700) and target censoring proportions (0.0 vs. 0.3). Panels correspond to different coefficient patterns. The dashed horizontal line indicates the nominal 90% level. All panels show coverage results for p=20.

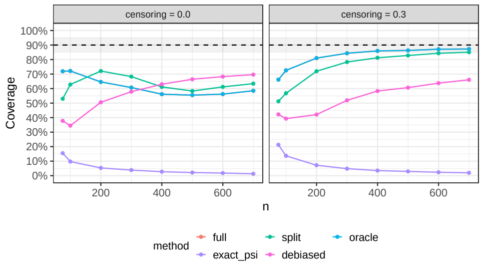

(a) All-ones pattern.

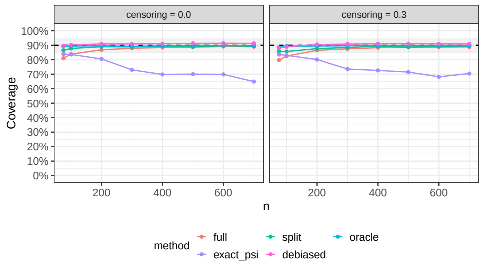

(b) High-contrast pattern.

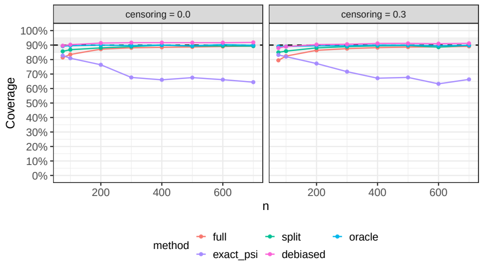

(c) Realistic pattern.

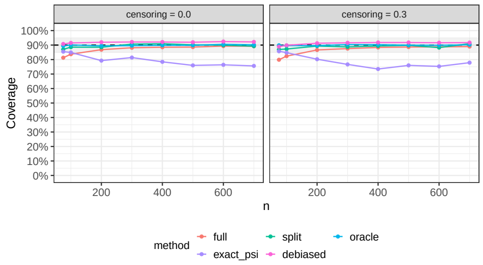

(d) Sparse pattern.

Fig. S6: Post-selection coverage in the toy simulations (simple setting) for the tuning choice $\lambda_{\mathrm{CV,1se}}$ across sample sizes (n = 75–700) and target censoring proportions (0.0 vs. 0.3). Panels correspond to different coefficient patterns. The dashed horizontal line indicates the nominal 90% level.All panels show coverage results for p=20.

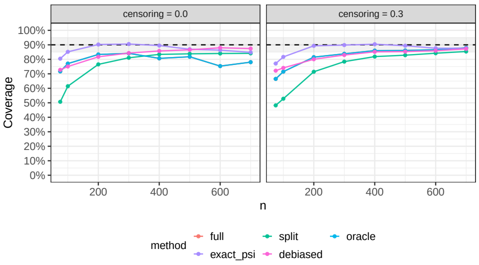

(a) All-ones pattern.

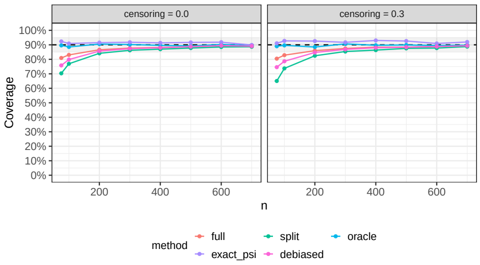

(b) High-contrast pattern.

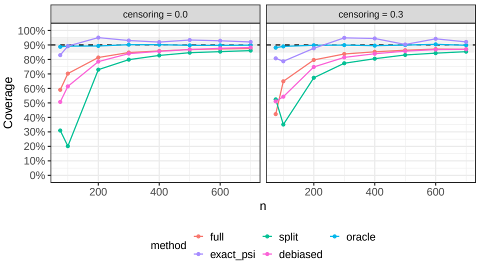

(c) Realistic pattern.

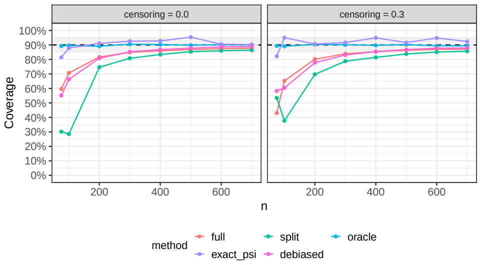

(d) Sparse pattern.

Fig. S7: Post-selection coverage in the toy simulations (simple setting) for a fixed tuning choice of λ (λ_{fix}) across sample sizes ($n = 75$–700) and target censoring proportions (0.0 vs. 0.3). Panels correspond to different coefficient patterns. The dashed horizontal line indicates the nominal 90% level. Panels a) and b) correspond to p=20. Panels c) and d) correspond to p=50.

S2.1.2 METABRIC simulation settings

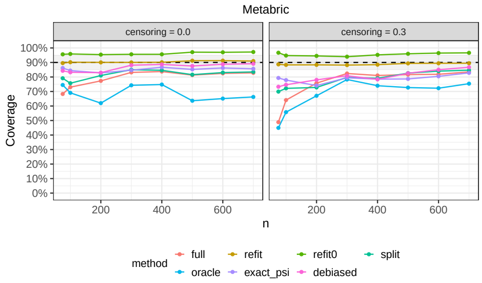

(a) $\lambda_{\mathrm{CV,min}}$.

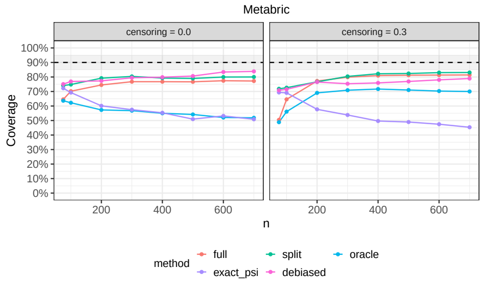

(b) $\lambda_{\mathrm{CV,1se}}$.

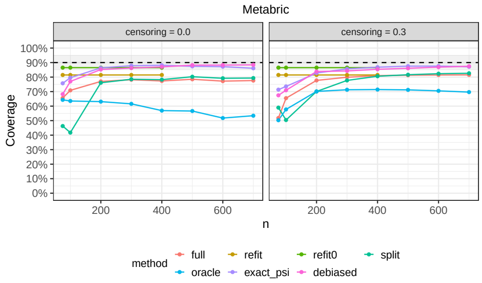

(c) λ_{AIC}.

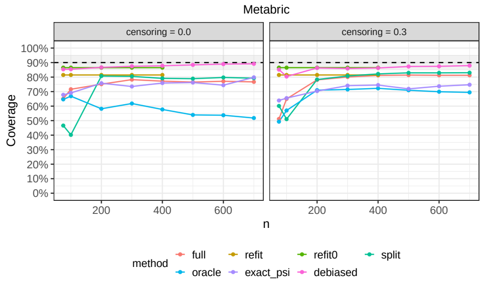

(d) λ_{BIC}.

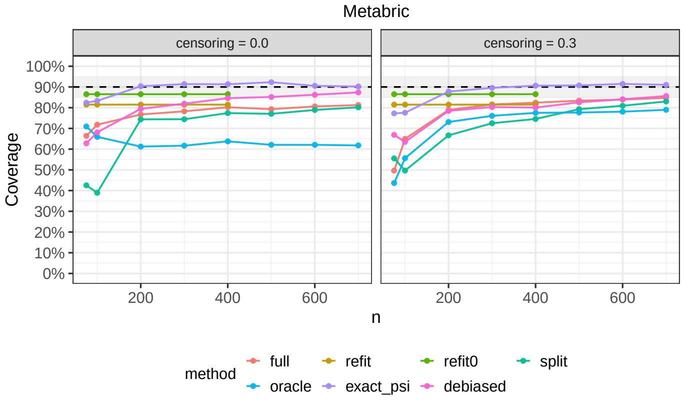

(e) λ_{fix}.

Fig. S8: Coverage of reported confidence intervals in the METABRIC simulation setting across sample sizes ($n = 75$–700) and target censoring proportions (0.0 vs. 0.3). Each panel corresponds to one tuning choice for λ (CV-based: $\lambda_{\mathrm{CV,min}}$, $\lambda_{\mathrm{CV,1se}}$; information-based: λ_{AIC}, λ_{BIC}; and λ_{fix}). The dashed horizontal line indicates the nominal 90% level. Oracle intervals are based on standard Cox Wald inference under the true active set and should therefore be interpreted as an asymptotic benchmark rather than as an exact finite-sample reference.

In the METABRIC simulation setting, oracle coverage occasionally departs from the nominal level, particularly at smaller sample sizes and under censoring. Since the oracle model uses the true active set, these deviations are not due to variable-selection error, but reflect finite-sample limitations of standard Cox Wald intervals in the calibrated METABRIC design. The oracle is therefore interpreted as a benchmark for knowing the active set, rather than as an exact finite-sample reference with guaranteed nominal coverage.

S2.1.3 Exploratory larger-candidate-set sensitivity analysis ($p = 100$)

To address the dimensionality concern raised during review without changing the primary scope of the study, we additionally performed a small exploratory sensitivity analysis with $p = 100$ candidate variables. These results are reported descriptively only. The setting still contains only a few signal variables and is therefore a sparse screening problem in which variable recovery can strongly affect all subsequent post-selection operating characteristics. It should not be interpreted as a comprehensive benchmark of very-high-dimensional Lasso screening.

Because coverage in this larger-candidate setting was strongly affected by the signal configuration and by tuning, we report the signal-term coverage results separately for each coefficient pattern. The panels vary the tuning rule, target censoring proportion, and correlation level, and the curves show the empirical coverage across sample sizes. These figures should be interpreted as sensitivity analyses for a harder screening problem rather than as part of the primary simulation grid.

The exploratory $p = 100$ results show substantial setting dependence. In coefficient patterns with clearer signal separation, several submodel-based procedures approach the nominal level in larger samples under some tuning rules, whereas coverage remains lower or more unstable in settings where signal recovery is more difficult. The allones pattern is particularly challenging because the information is spread across weak effects. The power and type I error summaries below are therefore interpreted jointly with the coverage and selection behavior, and not as target-invariant method rankings.

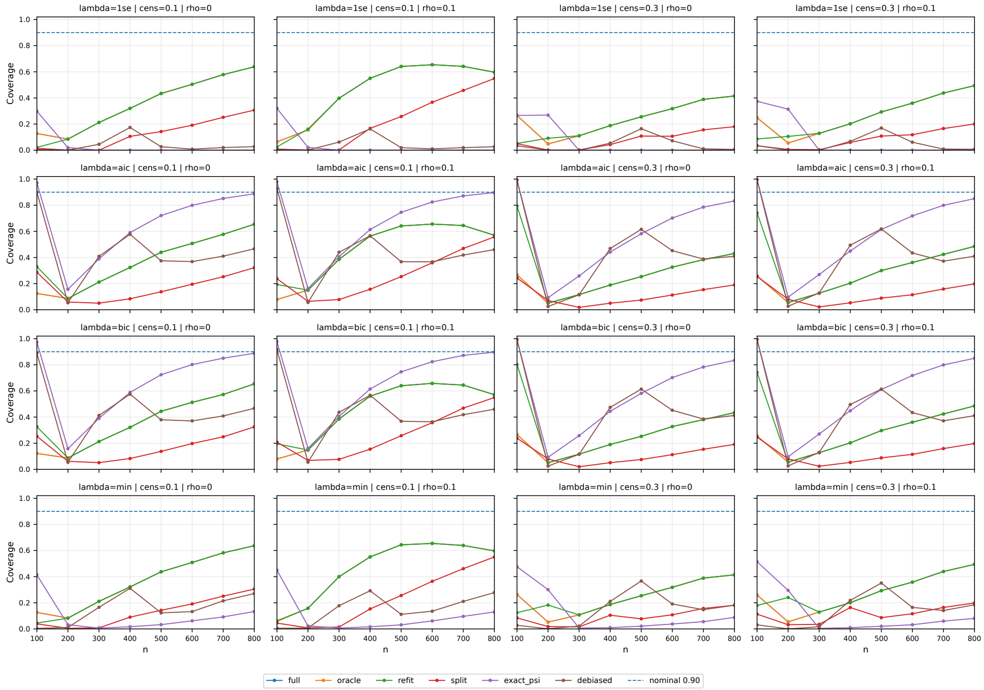

Fig. S9: Exploratory $p = 100$ sensitivity analysis: empirical signal-term coverage for the allones coefficient pattern. Panels are stratified by tuning rule, target censoring proportion, and correlation level. The dashed line indicates the nominal 90% level.

Table S1: Exploratory $p = 100$ sensitivity analysis at $n = 800$. Coverage summarizes empirical signal-term coverage averaged across the exploratory $p = 100$ coverage settings. Power and type I error are retained as descriptive summaries and should not be interpreted independently of the screening difficulty and method-specific targets.

Method	Signal-term coverage	Power	Type I error
full	0.575	1.000	0.135
oracle	0.845	1.000	–
refit	0.759	1.000	0.593
split	0.800	1.000	0.118
exact_psi	0.313	0.993	0.042
debiased	0.717	1.000	0.074

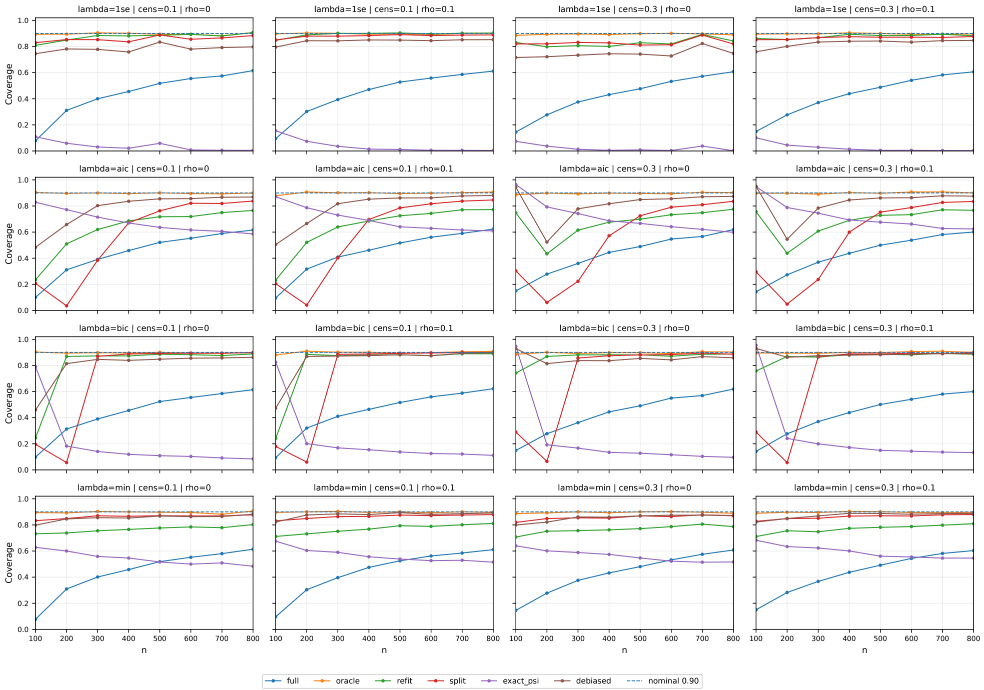

Fig. S10: Exploratory $p = 100$ sensitivity analysis: empirical signal-term coverage for the highcontrast coefficient pattern. Panels are stratified by tuning rule, target censoring proportion, and correlation level. The dashed line indicates the nominal 90% level.

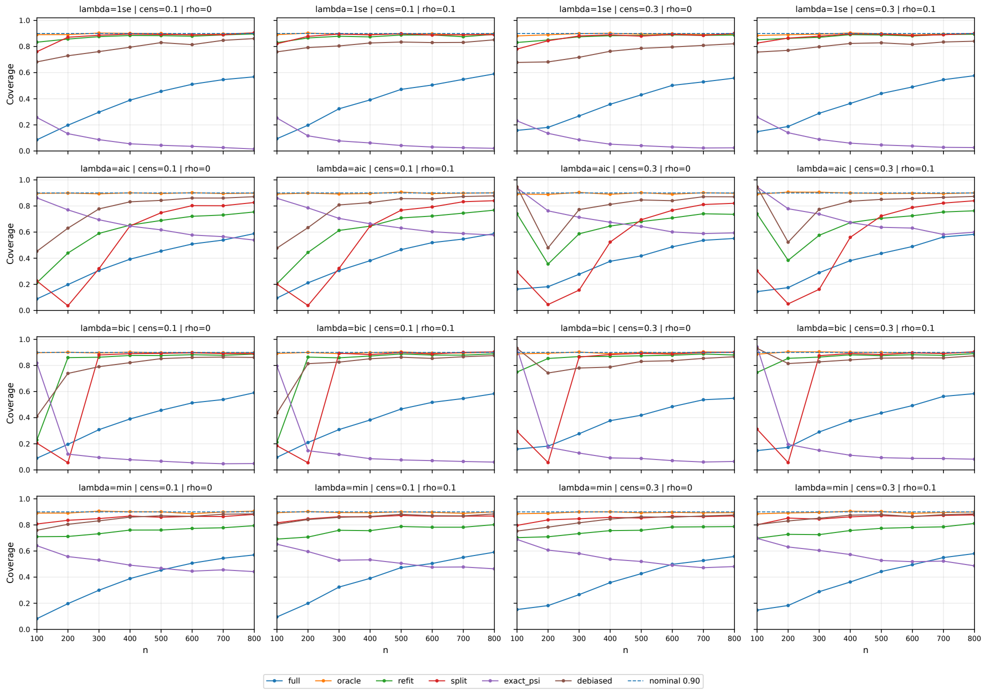

Fig. S11: Exploratory $p = 100$ sensitivity analysis: empirical signal-term coverage for the realistic coefficient pattern. Panels are stratified by tuning rule, target censoring proportion, and correlation level. The dashed line indicates the nominal 90% level.

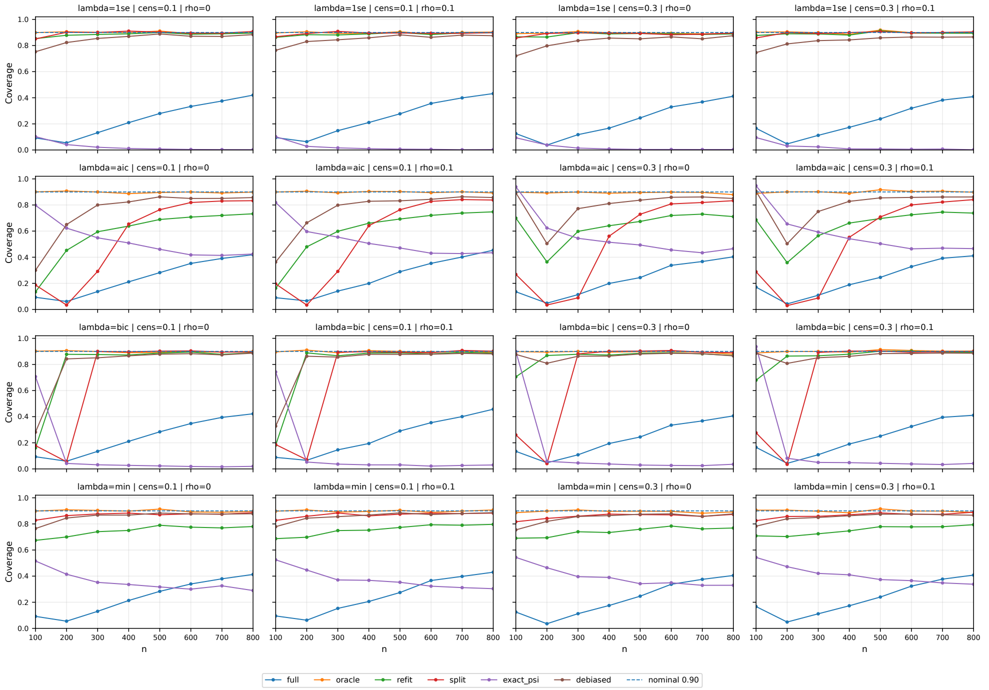

Fig. S12: Exploratory $p = 100$ sensitivity analysis: empirical signal-term coverage for the sparse coefficient pattern. Panels are stratified by tuning rule, target censoring proportion, and correlation level. The dashed line indicates the nominal 90% level.

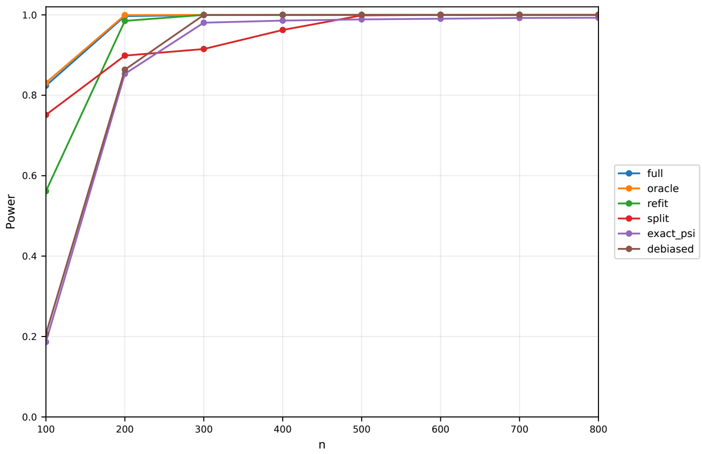

(a) Power.

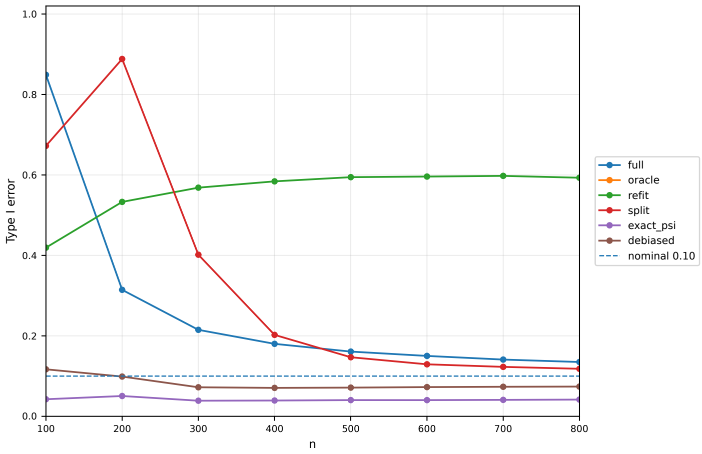

(b) Type I error.

Fig. S13: Exploratory $p = 100$ larger-candidate-set sensitivity analysis. Panels show empirical power and type I error by sample size and method. These operating characteristics are interpreted descriptively and jointly with the signal-term coverage results in Figures S9–S12.

S2.2 Confidence interval length

This subsection summarizes the distribution of confidence interval lengths for the core methods considered in the simulation study, focusing on their variability and relative efficiency across sample sizes.

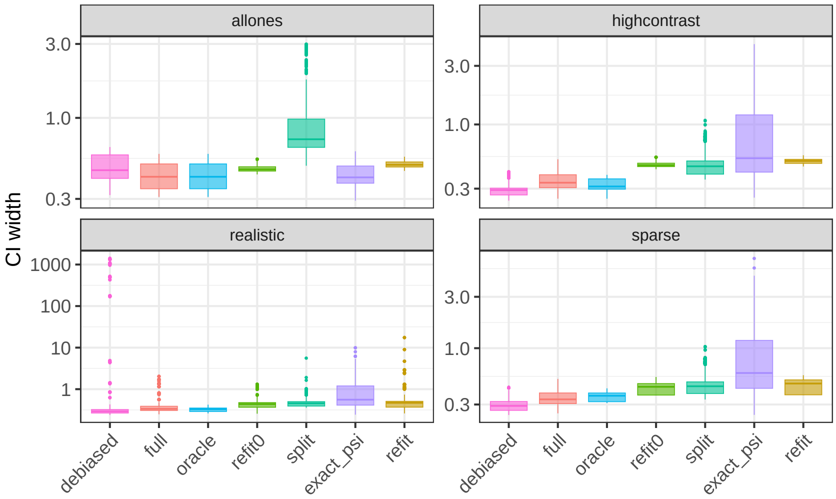

(a) Log scale.

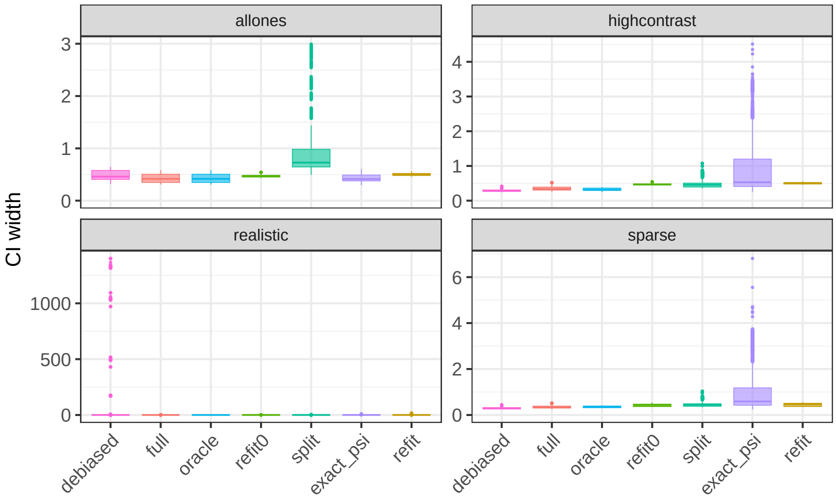

(b) Truncated y-axis.

Fig. S14: CI length distributions across core methods for $n = 200$: (a) log scale and (b) truncated y-axis.

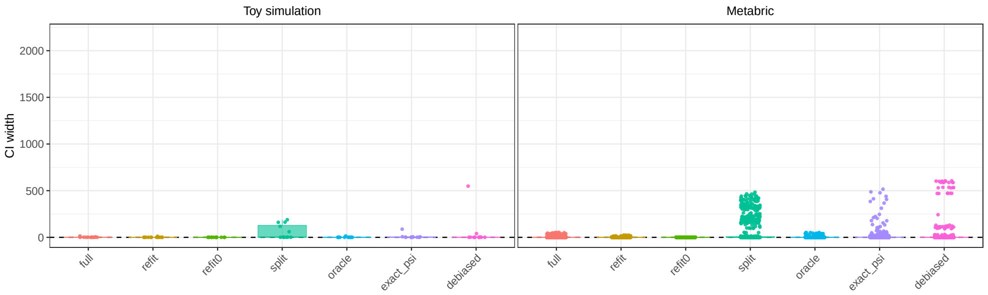

(a) $n = 75$

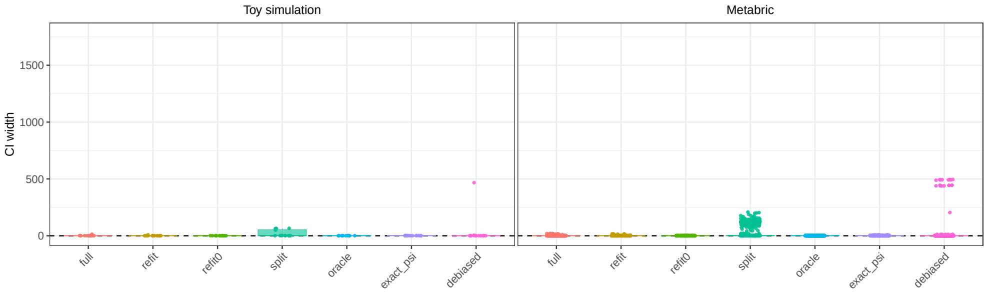

(b) $n = 100$

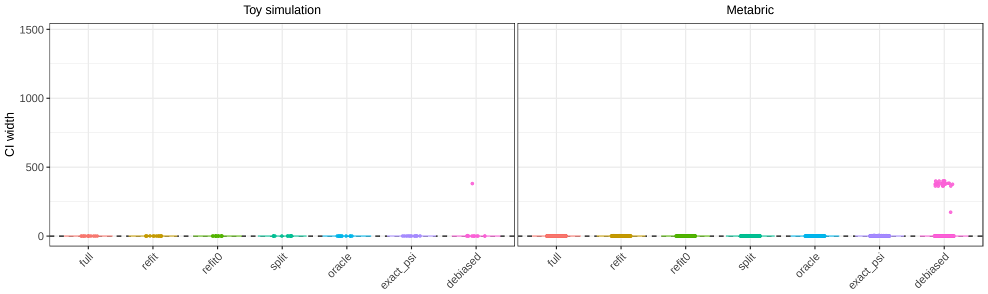

(c) $n = 200$

Fig. S15: Distribution of confidence interval lengths across core methods for different sample sizes. Panels show results for (a) $n = 75$, (b) $n = 100$, and (c) $n = 200$.

S2.3 Post-selection power and post-selection type-I-error results

This subsection presents post-selection power and post-selection type I error rates for the considered inference procedures. Results are shown for different sample sizes and tuning parameter choices to illustrate the trade-off between power and error control.

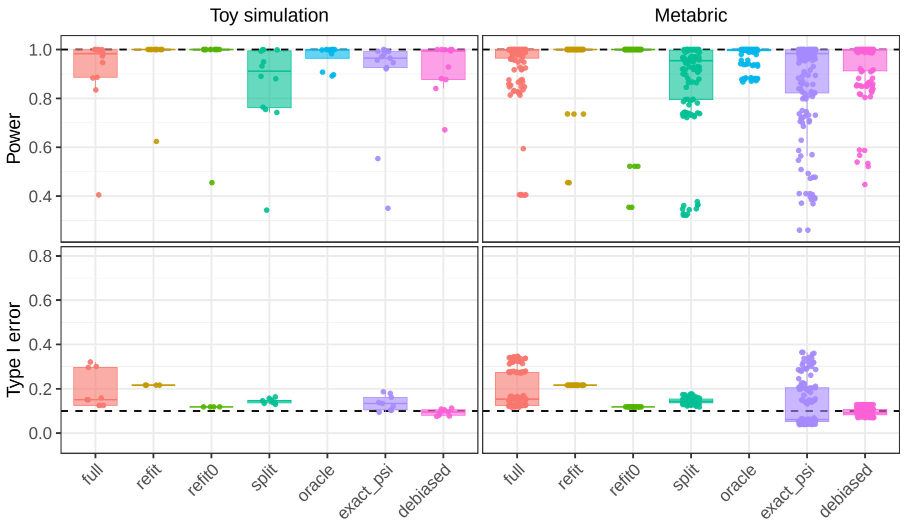

(a) $n = 100$

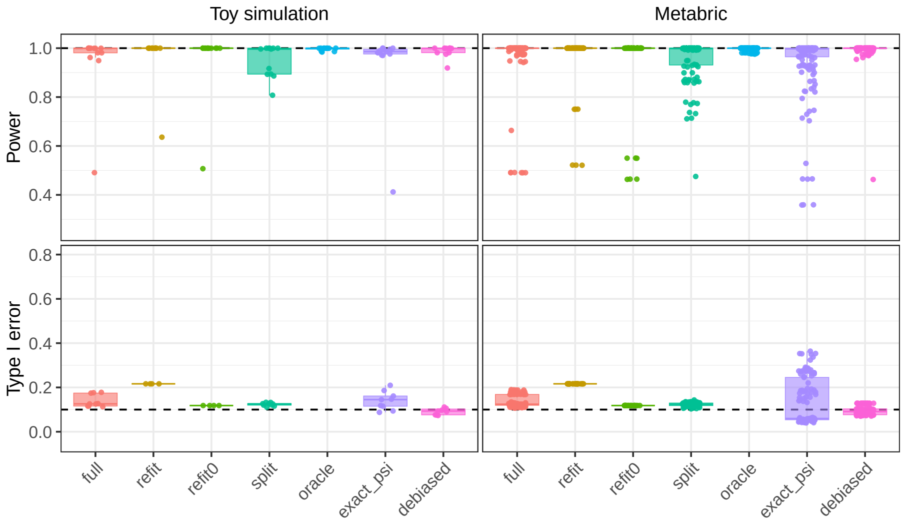

(b) $n = 200$

Fig. S16: Post-selection power and post-selection type I error rates under the toy and metabric settings. Panels show results for (a) $n = 100$ and (b) $n = 200$ across inference methods for the penalty tuning choice $\lambda_{\mathrm{CV,min}}$; the dashed horizontal line indicates the nominal significance level.

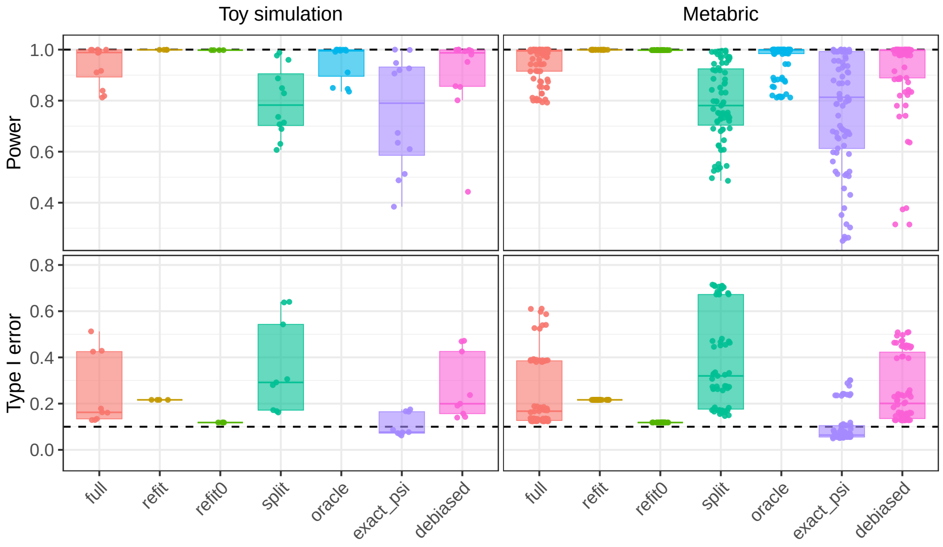

(a) $n = 75$

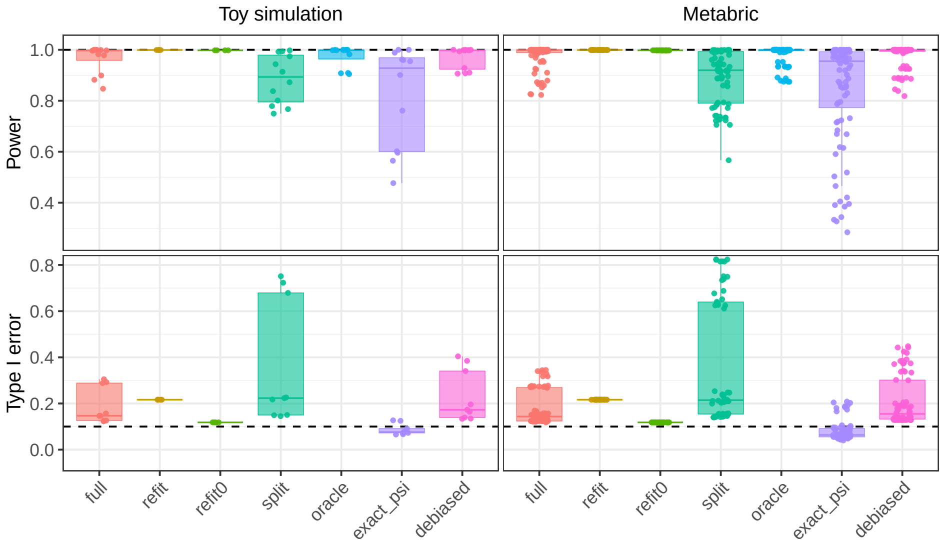

(b) $n = 100$

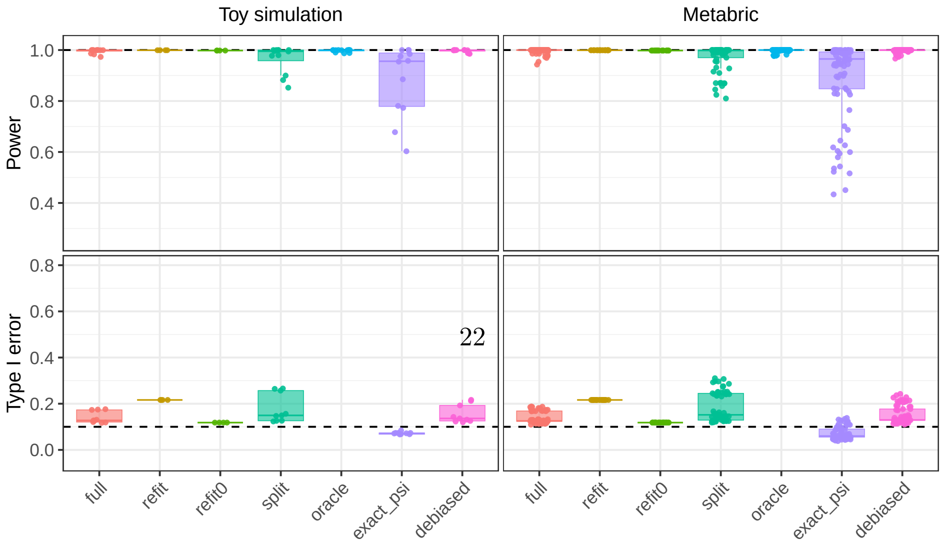

(c) $n = 200$

Fig. S17: Post-selection power and post-selection type I error rates under the toy and metabric settings for the one-standard-error tuning choice λ_{fix}. Panels show results for (a) $n = 75$, (b) $n = 100$, and (c) $n = 200$.

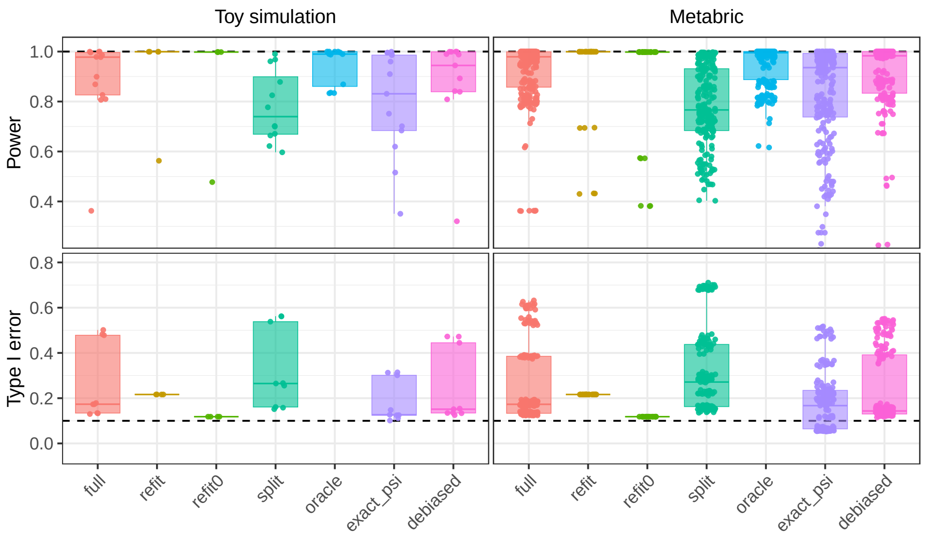

(a) $n = 75$

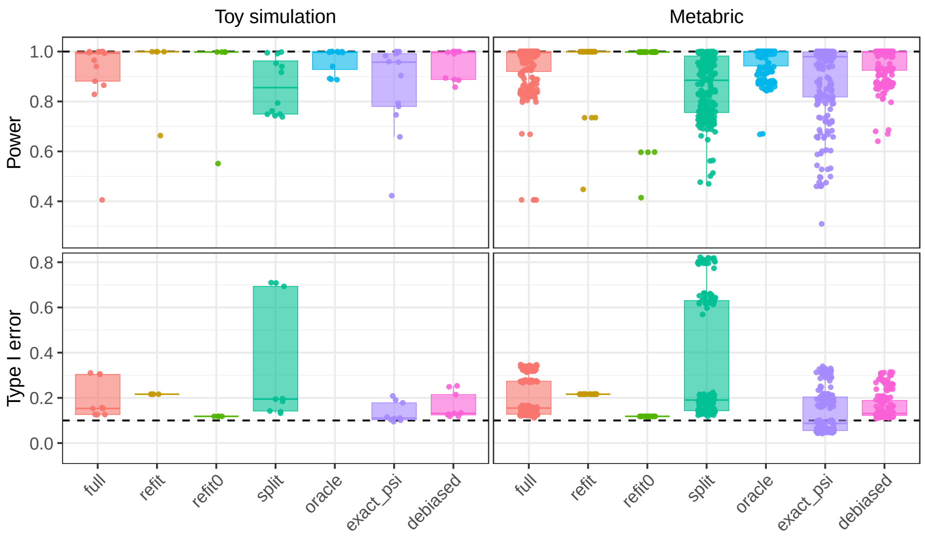

(b) $n = 100$

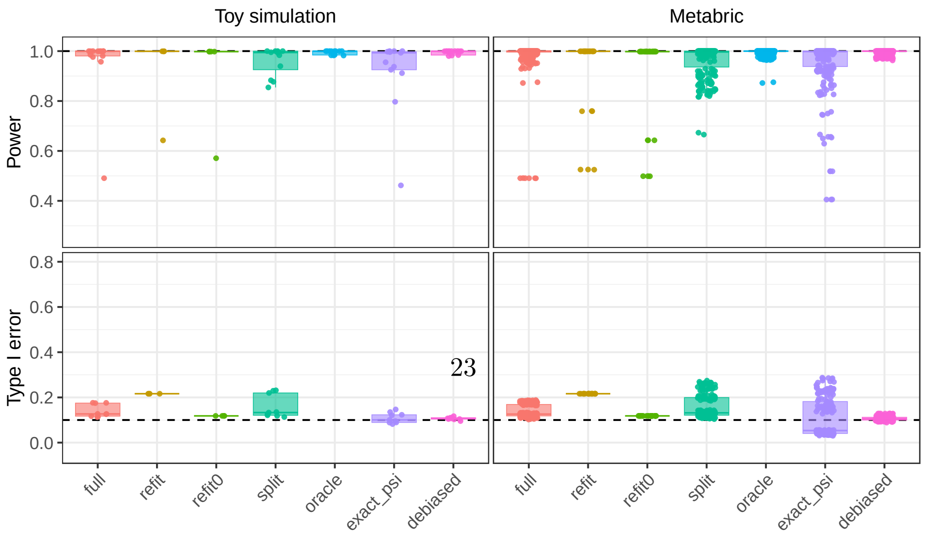

(c) $n = 200$

Fig. S18: Post-selection power and post-selection type I error rates under the toy and metabric settings for the AIC-based tuning choice λ_{AIC}. Panels show results for (a) $n = 75$, (b) $n = 100$, and (c) $n = 200$.

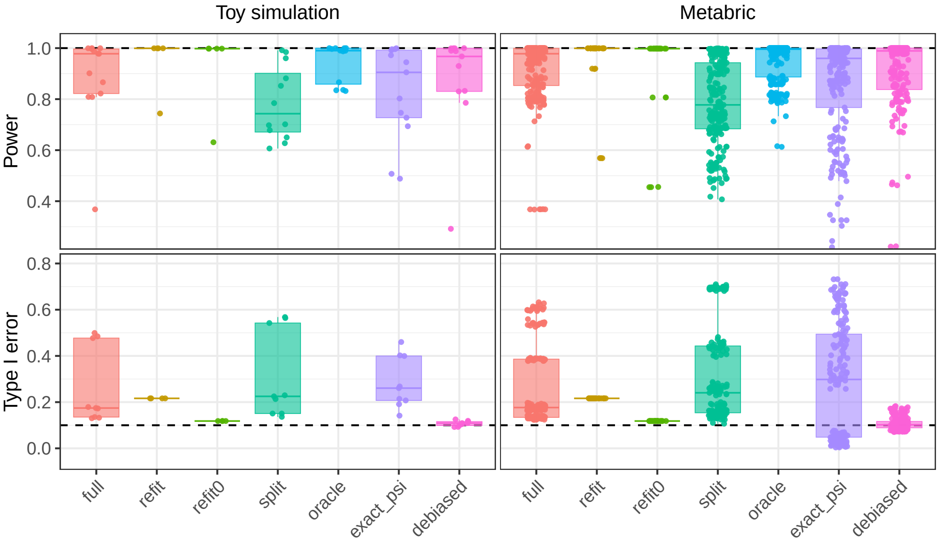

(a) $n = 75$

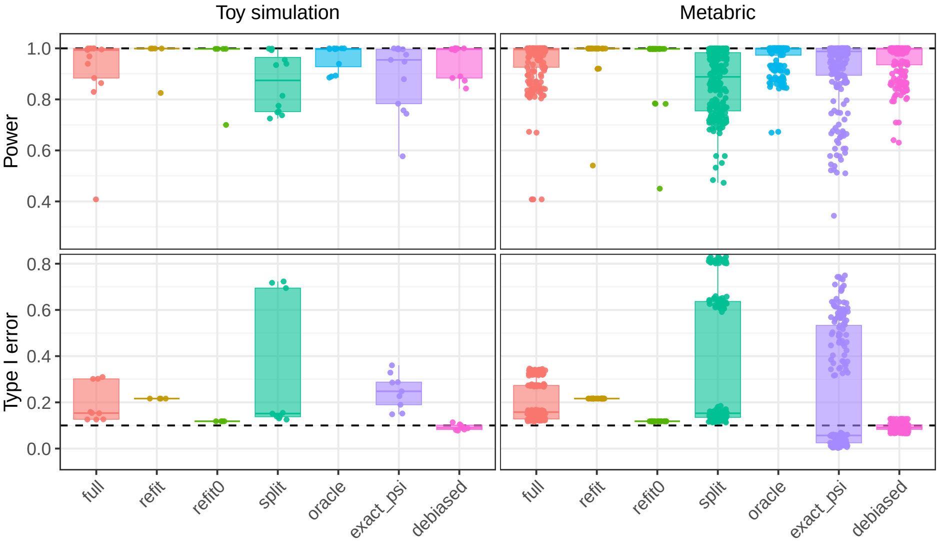

(b) $n = 100$

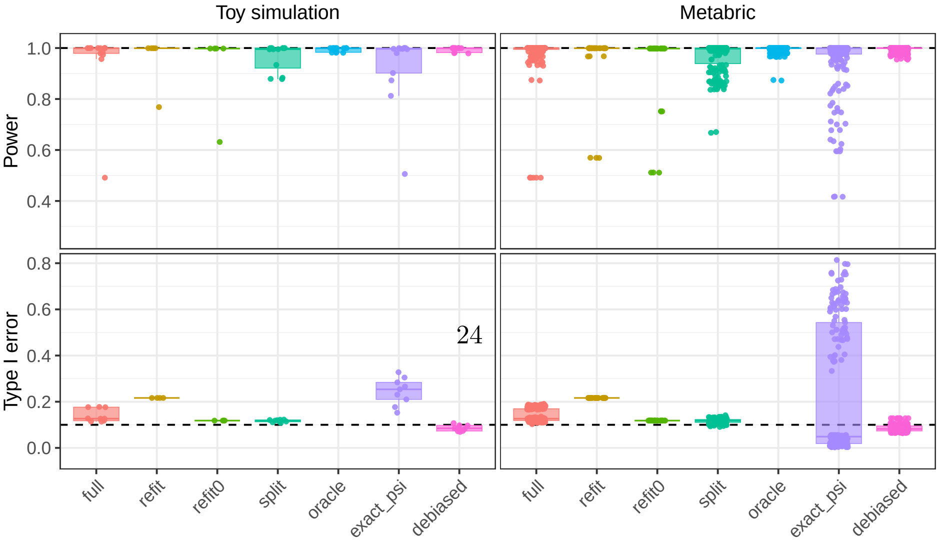

(c) $n = 200$

Fig. S19: Post-selection power and post-selection type I error rates under the toy and metabric settings for the BIC-based tuning choice λ_{BIC}. Panels show results for (a) $n = 75$, (b) $n = 100$, and (c) $n = 200$.

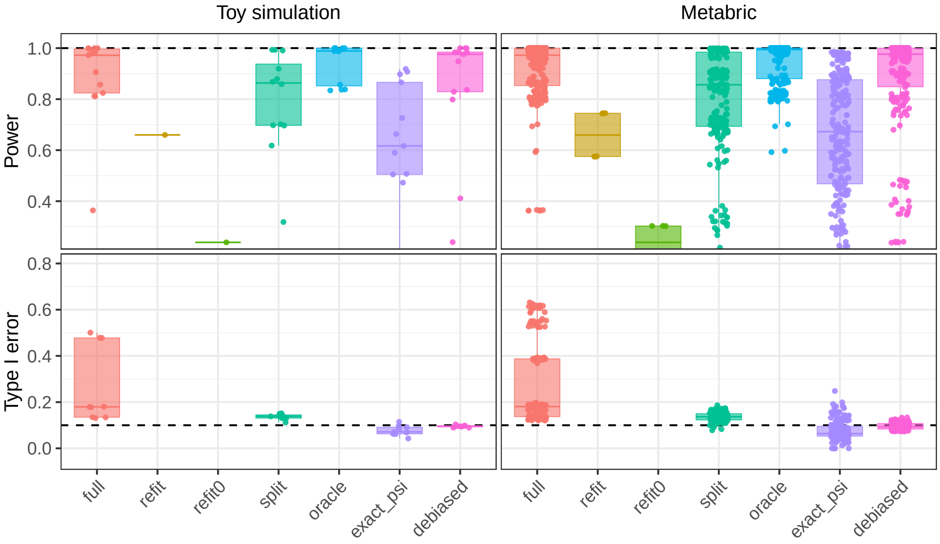

(a) $n = 75$

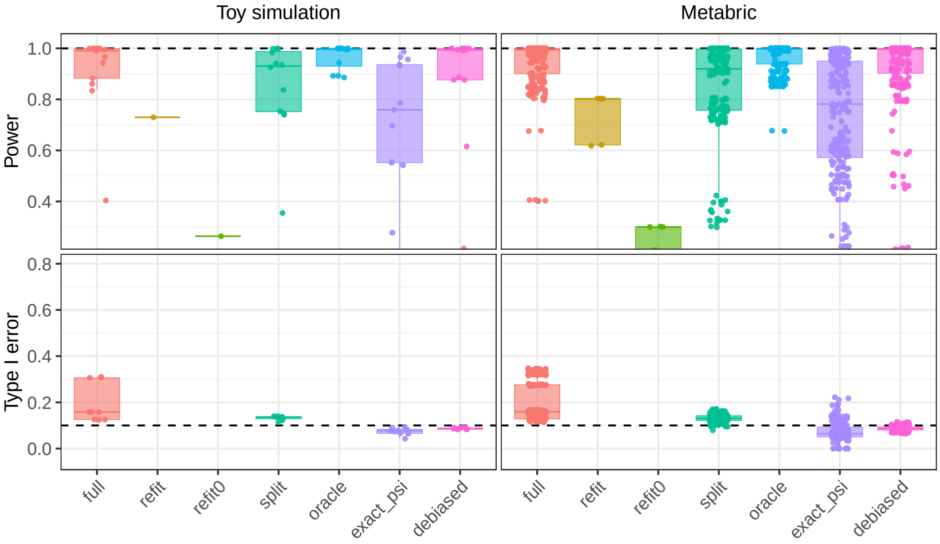

(b) $n = 100$

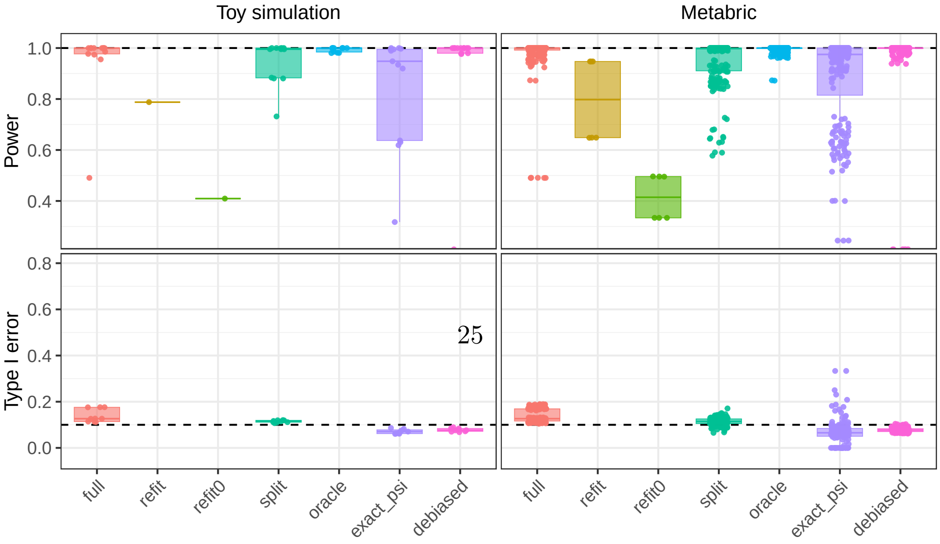

(c) $n = 200$

Fig. S20: Post-selection power and post-selection type I error rates under the toy and metabric settings for the fixed tuning choice $\lambda_{\mathrm{CV,1se}}$. Panels show results for (a) $n = 75$, (b) $n = 100$, and (c) $n = 200$.

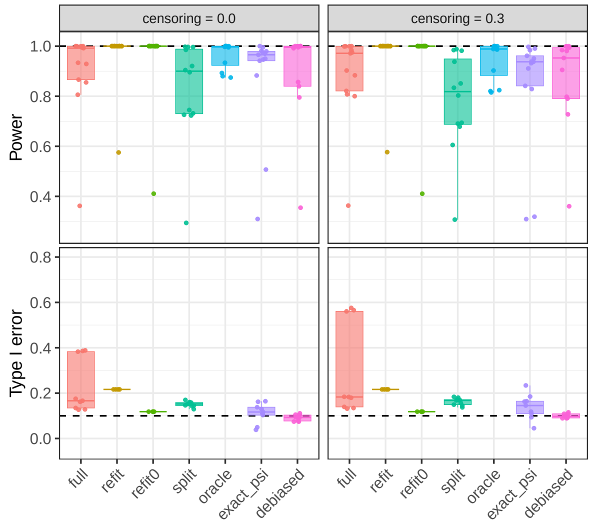

(a) $n = 75$

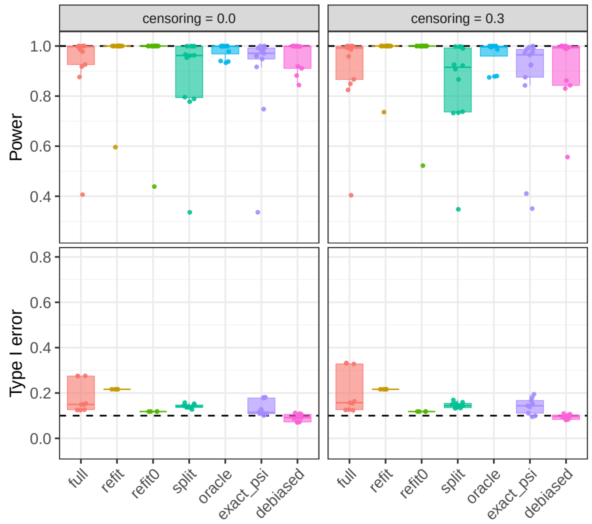

(b) $n = 100$

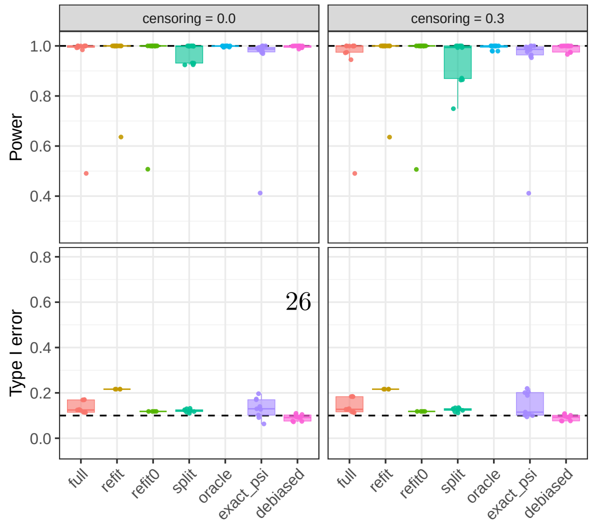

(c) $n = 200$

Fig. S21: Post-selection power and post-selection type I error rates under the toy and metabric settings for the $\lambda_{\text{CV,min}}$ tuning choice. Panels show (a) $n = 75$, (b) $n = 100$, and (c) $n = 200$.

S2.3.1 Post-selection power and post-selection type-I error results by target censoring - toy simulation setting

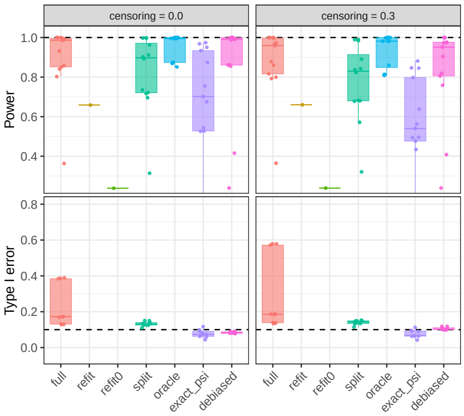

(a) $n = 75$

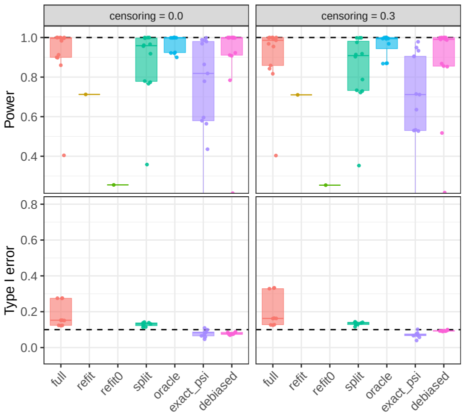

(b) $n = 100$

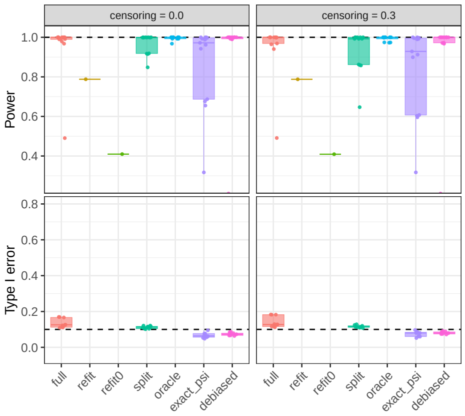

(c) $n = 200$

Fig. S22: Post-selection power and post-selection type I error rates under the toy setting for the one-standard-error tuning choice $\lambda_{\text{CV,1se}}$, stratified by target censoring proportion. Panels show results for (a) $n = 75$, (b) $n = 100$, and (c) $n = 200$.

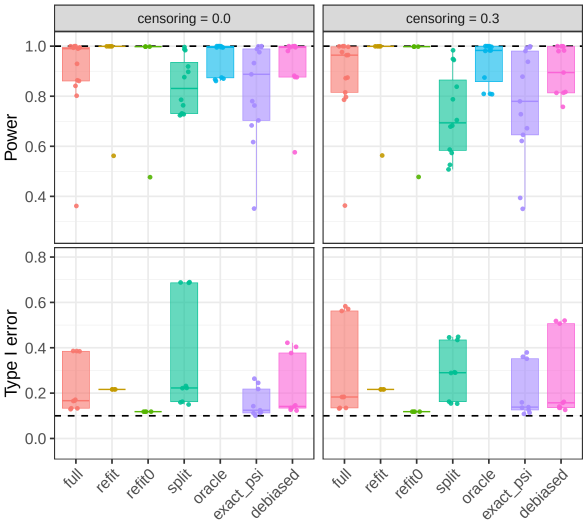

(a) $n = 75$

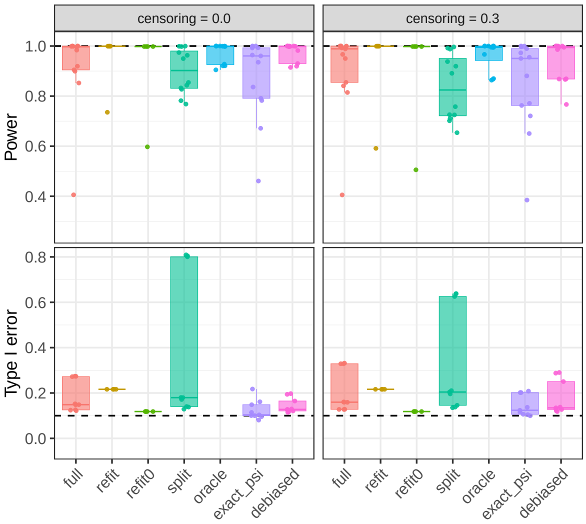

(b) $n = 100$

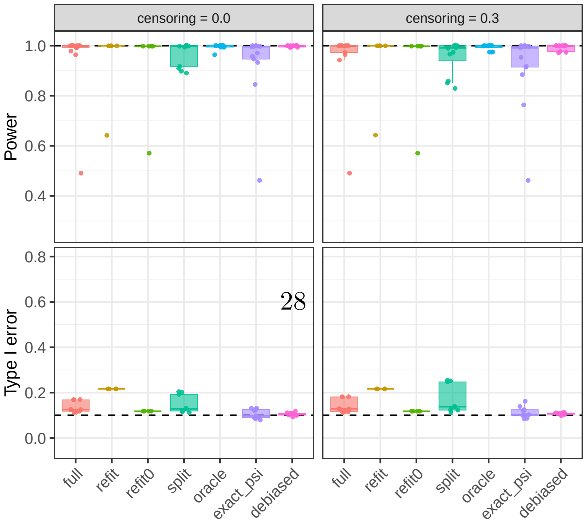

(c) $n = 200$

Fig. S23: Post-selection power and post-selection type I error rates under the toy setting for the AIC-based tuning choice λ_{AIC}, stratified by target censoring proportion. Panels show results for (a) $n = 75$, (b) $n = 100$, and (c) $n = 200$.

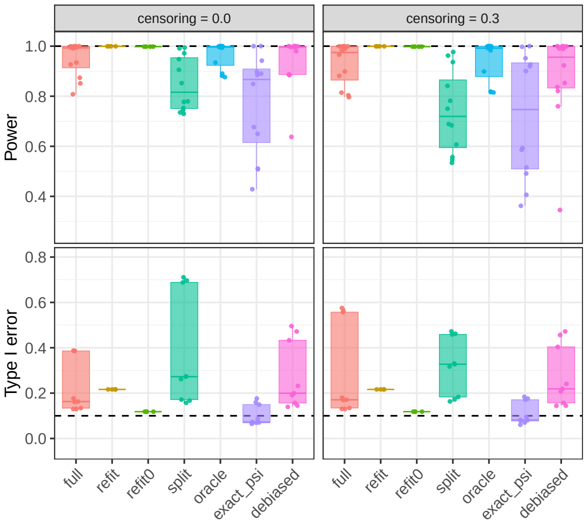

(a) $n = 75$

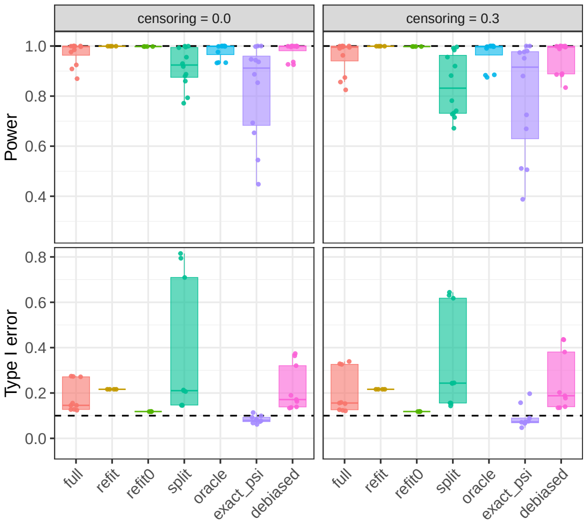

(b) $n = 100$

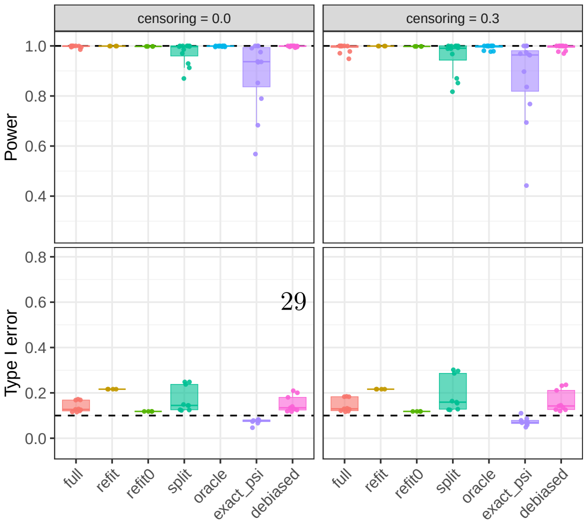

(c) $n = 200$

Fig. S24: Post-selection power and post-selection type I error rates under the toy simulation setting for the fixed tuning choice λ_{fix}, stratified by target censoring proportion. Panels show results for (a) $n = 75$, (b) $n = 100$, and (c) $n = 200$.

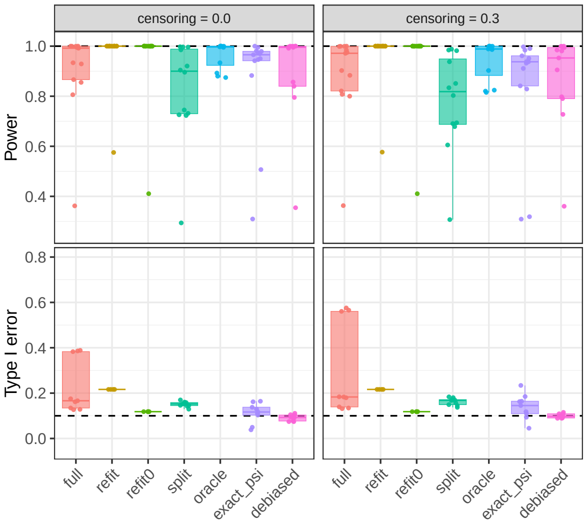

(a) $n = 75$

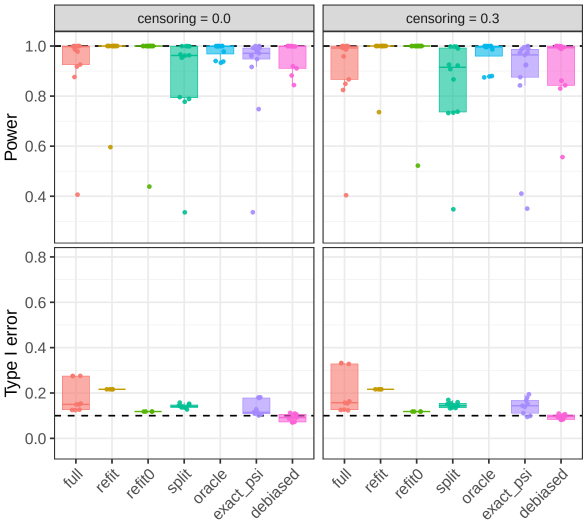

(b) $n = 100$

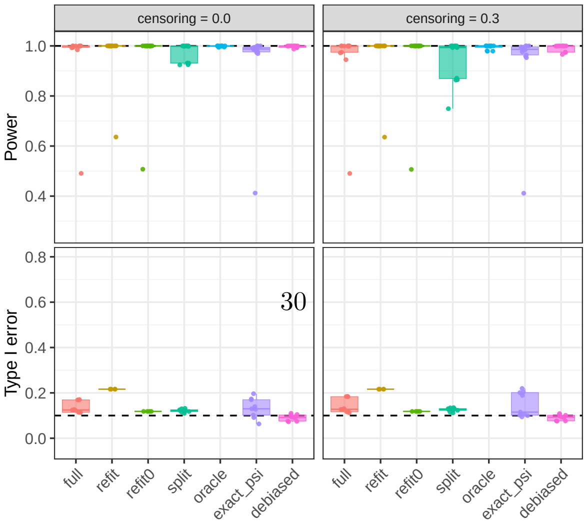

(c) $n = 200$

Fig. S25: Post-selection power and post-selection type I error rates under the toy simulation setting for the $\lambda_{\text{cv,min}}$ tuning choice, stratified by target censoring proportion. Panels show results for (a) $n = 75$, (b) $n = 100$, and (c) $n = 200$.

S2.3.2 Post-selection power and post-selection type-I error results by target censoring - metabric simulation setting

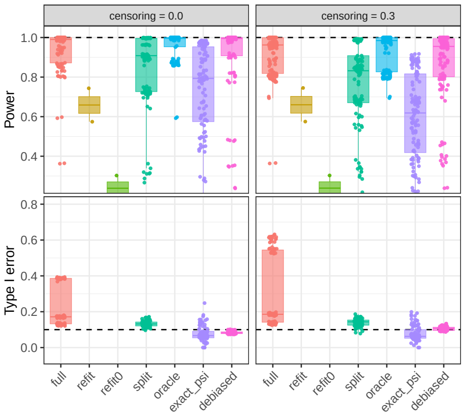

(a) $n = 75$

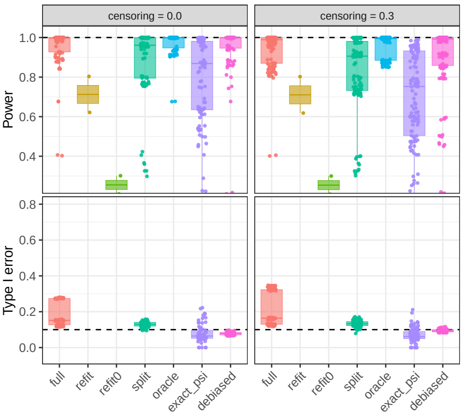

(b) $n = 100$

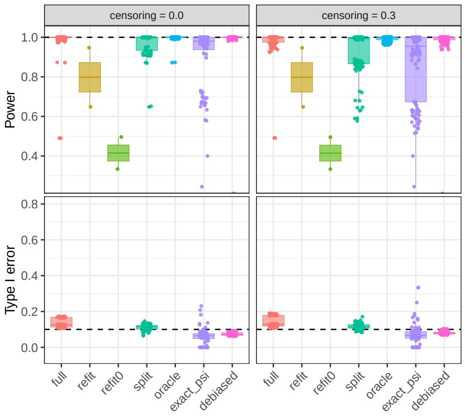

(c) $n = 200$

Fig. S26: Post-selection power and post-selection type I error rates under the metabric simulation setting for the one-standard-error tuning choice $\lambda_{\text{CV},1\text{se}}$, stratified by target censoring proportion. Panels show results for (a) $n = 75$, (b) $n = 100$, and (c) $n = 200$.

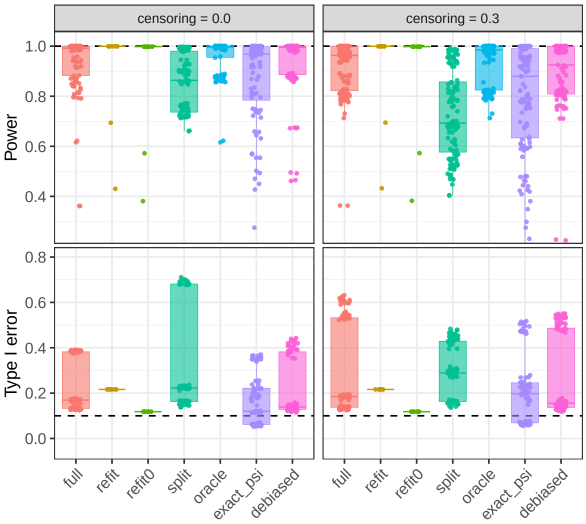

(a) $n = 75$

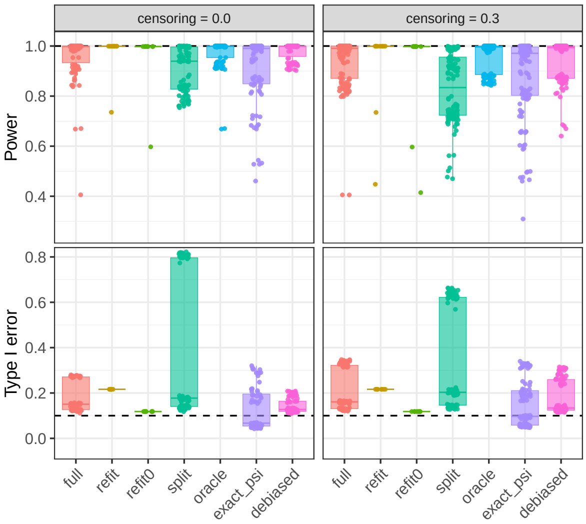

(b) $n = 100$

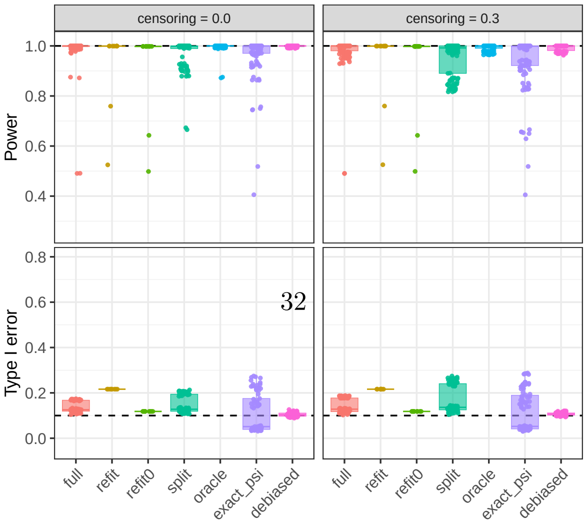

(c) $n = 200$

Fig. S27: Post-selection power and post-selection type I error rates under the metabric simulation setting for the AIC-based tuning choice λ_{AIC}, stratified by target censoring proportion. Panels show results for (a) $n = 75$, (b) $n = 100$, and (c) $n = 200$.

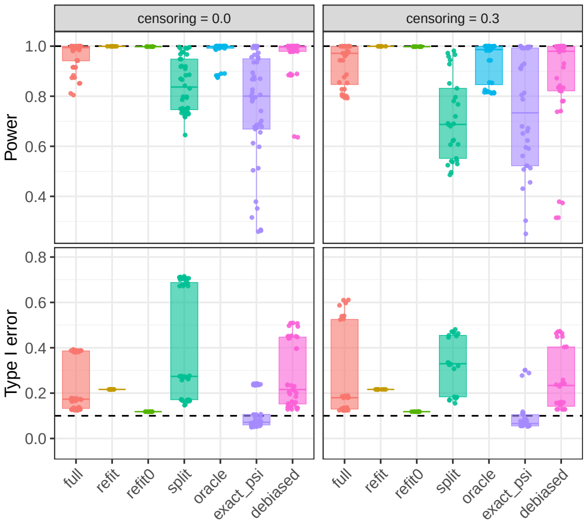

(a) $n = 75$

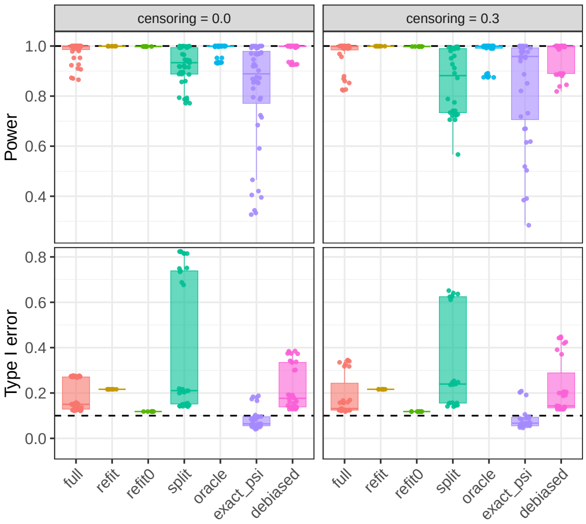

(b) $n = 100$

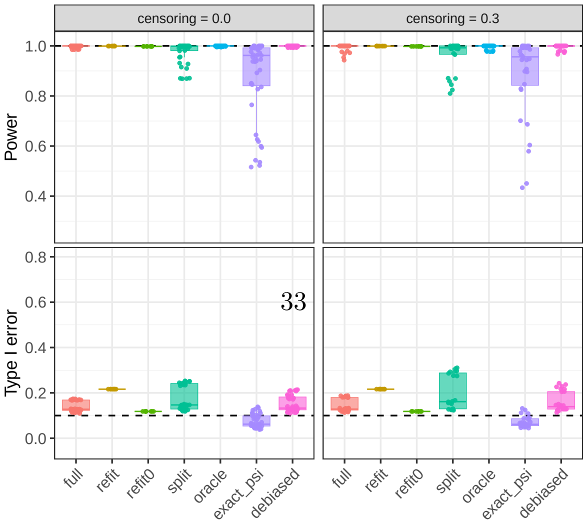

(c) $n = 200$

Fig. S28: Post-selection power and post-selection type I error rates under the metabric simulation setting for the fixed tuning choice λ_{fix}, stratified by target censoring proportion. Panels show results for (a) $n = 75$, (b) $n = 100$, and (c) $n = 200$.

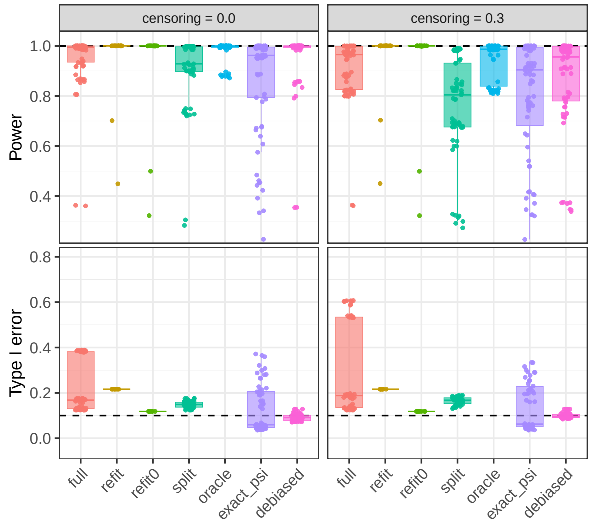

(a) $n = 75$

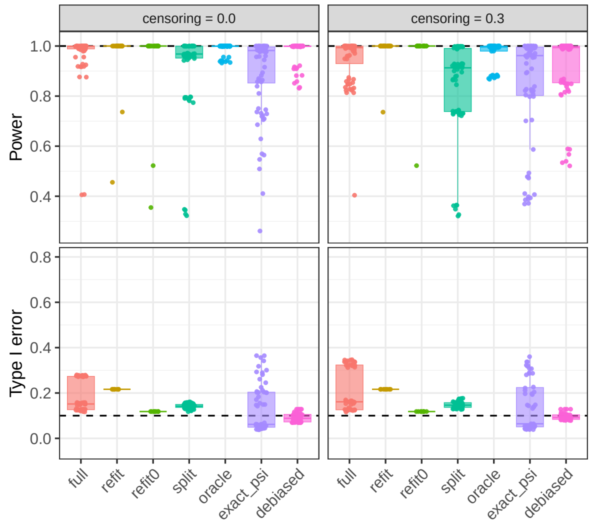

(b) $n = 100$

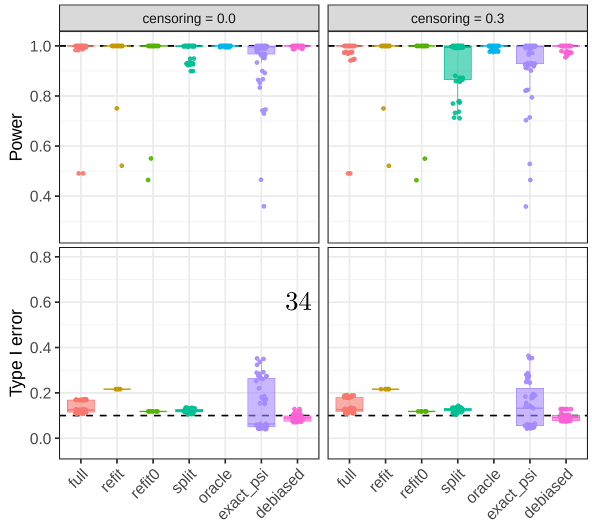

(c) $n = 200$

Fig. S29: Post-selection power and post-selection type I error rates under the metabric simulation setting for the $\lambda_{\mathrm{CV,min}}$ tuning choice, stratified by target censoring proportion. Panels show results for (a) $n = 75$, (b) $n = 100$, and (c) $n = 200$.

S2.4 Runtimes

This subsection reports average computational runtimes for the core methods, highlighting their relative computational efficiency under representative simulation scenarios.

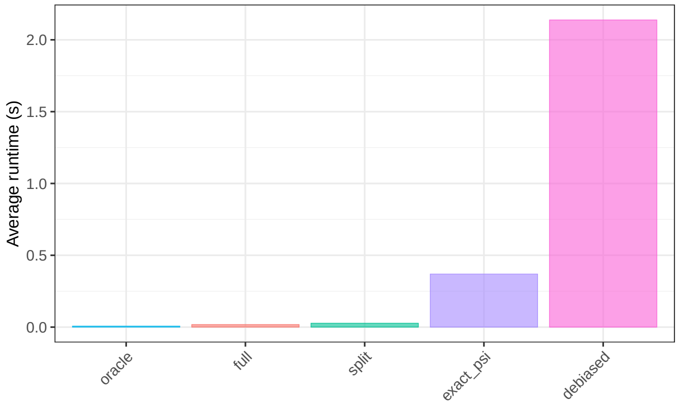

Fig. S30: Average computational runtimes (seconds) for the core methods at sample size $n = 200$, averaged over toy simulation scenarios.

S2.5 Performance measures

Table S2: Predictive and selection performance for $n = 200$, $p = 10$ (exponential baseline). Entries are mean (SE) for IBS and mean for P_{true} and model size.

	$\pi_C = 0.0$			$\pi_C = 0.1$			$\pi_C = 0.3$		
Method	IBS	P_{true}	size	IBS	P_{true}	size	IBS	P_{true}	size
Correlation $\rho = 0.0$									
LASSO-1se	0.261 (0.004)	0.65	4.5	0.127 (0.000)	0.70	4.4	0.141 (0.000)	0.75	4.3
LASSO-aic	0.225 (0.004)	0.04	7.5	0.115 (0.000)	0.03	7.4	0.128 (0.000)	0.03	7.4
LASSO-bic	0.229 (0.004)	0.25	5.6	0.117 (0.000)	0.25	5.6	0.131 (0.000)	0.24	5.6
LASSO-min	0.226 (0.004)	0.03	7.3	0.117 (0.000)	0.04	7.2	0.130 (0.000)	0.03	7.2
Oracle	0.281 (0.003)	–	4.0	0.121 (0.000)	–	4.0	0.135 (0.001)	–	4.0
Correlation $\rho = 0.1$									
LASSO-1se	0.258 (0.004)	0.71	4.4	0.121 (0.000)	0.73	4.4	0.136 (0.000)	0.75	4.3
LASSO-aic	0.217 (0.004)	0.05	7.4	0.111 (0.000)	0.03	7.3	0.124 (0.000)	0.06	7.2
LASSO-bic	0.225 (0.004)	0.27	5.5	0.113 (0.000)	0.27	5.5	0.126 (0.000)	0.29	5.5
LASSO-min	0.223 (0.004)	0.03	7.1	0.111 (0.000)	0.03	7.2	0.125 (0.000)	0.04	7.1
Oracle	0.278 (0.003)	–	4.0	0.116 (0.000)	–	4.0	0.131 (0.001)	–	4.0

Table S3: Predictive and selection performance for $n = 200$, $p = 10$ (Weibull baseline). Entries are mean (SE) for IBS and mean for P_{true} and model size.

	$\pi_C = 0.0$			$\pi_C = 0.1$			$\pi_C = 0.3$		
Method	IBS	P_{true}	size	IBS	P_{true}	size	IBS	P_{true}	size
Correlation $\rho = 0.0$									
LASSO-1se	0.227 (0.003)	0.65	4.5	0.127 (0.000)	0.69	4.4	0.132 (0.000)	0.69	4.4
LASSO-aic	0.210 (0.003)	0.04	7.4	0.118 (0.000)	0.04	7.4	0.121 (0.000)	0.05	7.4
LASSO-bic	0.215 (0.003)	0.26	5.6	0.119 (0.000)	0.23	5.6	0.123 (0.000)	0.23	5.6
LASSO-min	0.206 (0.003)	0.02	7.3	0.119 (0.000)	0.04	7.2	0.122 (0.000)	0.02	7.2
Oracle	0.255 (0.082)	–	4.0	0.122 (0.000)	–	4.0	0.127 (0.000)	–	4.0
Correlation $\rho = 0.1$									
LASSO-1se	0.219 (0.003)	0.72	4.4	0.123 (0.000)	0.72	4.4	0.129 (0.000)	0.75	4.3
LASSO-aic	0.197 (0.003)	0.04	7.3	0.113 (0.000)	0.04	7.3	0.117 (0.000)	0.05	7.3
LASSO-bic	0.199 (0.003)	0.30	5.4	0.115 (0.000)	0.28	5.4	0.119 (0.000)	0.26	5.5
LASSO-min	0.202 (0.003)	0.03	7.2	0.113 (0.000)	0.03	7.2	0.119 (0.000)	0.04	7.1
Oracle	0.248 (0.003)	–	4.0	0.118 (0.000)	–	4.0	0.124 (0.000)	–	4.0

Table S4: Predictive and selection performance for $n = 200$, $p = 20$ (exponential baseline). Entries are mean (SE) for IBS and mean for P_{true} and model size.

	$\pi_C = 0.0$			$\pi_C = 0.1$			$\pi_C = 0.3$		
Method	IBS	P_{true}	size	IBS	P_{true}	size	IBS	P_{true}	size
Correlation $\rho = 0.0$									
LASSO-1se	0.251 (0.004)	0.51	4.9	0.127 (0.000)	0.53	4.8	0.143 (0.000)	0.60	4.7
LASSO-aic	0.225 (0.004)	0.01	11.4	0.114 (0.000)	0.01	11.5	0.126 (0.000)	0.01	11.3
LASSO-bic	0.243 (0.004)	0.21	6.1	0.120 (0.000)	0.21	6.1	0.132 (0.000)	0.19	6.2
LASSO-min	0.227 (0.004)	0.01	9.9	0.116 (0.000)	0.01	9.8	0.129 (0.000)	0.01	9.5
Oracle	0.283 (0.003)	–	4.0	0.121 (0.000)	–	4.0	0.135 (0.001)	–	4.0
Correlation $\rho = 0.1$									
LASSO-1se	0.257 (0.004)	0.55	4.8	0.123 (0.000)	0.61	4.6	0.136 (0.000)	0.66	4.5
LASSO-aic	0.221 (0.004)	0.01	11.4	0.109 (0.000)	0.01	11.2	0.122 (0.000)	0.02	11.0
LASSO-bic	0.235 (0.004)	0.23	5.9	0.115 (0.000)	0.25	5.8	0.127 (0.000)	0.25	5.9
LASSO-min	0.225 (0.004)	0.01	9.8	0.111 (0.000)	0.01	9.5	0.124 (0.000)	0.00	9.4
Oracle	0.277 (0.003)	–	4.0	0.116 (0.000)	–	4.0	0.130 (0.001)	–	4.0

Table S5: Predictive and selection performance for $n = 200$, $p = 20$ (Weibull baseline). Entries are mean (SE) for IBS and mean for P_{true} and model size.

	$\pi_C = 0.0$			$\pi_C = 0.1$			$\pi_C = 0.3$		
Method	IBS	P_{true}	size	IBS	P_{true}	size	IBS	P_{true}	size
Correlation $\rho = 0.0$									
LASSO-1se	0.233 (0.003)	0.47	5.0	0.128 (0.000)	0.50	4.9	0.134 (0.000)	0.56	4.8
LASSO-aic	0.204 (0.003)	0.01	11.5	0.116 (0.000)	0.01	11.6	0.119 (0.000)	0.01	11.3
LASSO-bic	0.217 (0.003)	0.23	5.9	0.121 (0.000)	0.21	6.0	0.125 (0.000)	0.21	6.2
LASSO-min	0.209 (0.003)	0.01	9.7	0.118 (0.000)	0.01	9.7	0.122 (0.000)	0.01	9.7
Oracle	0.254 (0.003)	–	4.0	0.122 (0.000)	–	4.0	0.127 (0.000)	–	4.0
Correlation $\rho = 0.1$									
LASSO-1se	0.224 (0.003)	0.54	4.9	0.124 (0.000)	0.58	4.7	0.130 (0.000)	0.61	4.7
LASSO-aic	0.198 (0.003)	0.02	11.3	0.111 (0.000)	0.01	11.0	0.116 (0.000)	0.02	10.8
LASSO-bic	0.206 (0.003)	0.23	6.0	0.117 (0.000)	0.25	5.9	0.121 (0.000)	0.23	6.0
LASSO-min	0.205 (0.003)	0.01	9.5	0.114 (0.000)	0.01	9.5	0.118 (0.000)	0.02	9.3
Oracle	0.251 (0.002)	–	4.0	0.118 (0.000)	–	4.0	0.123 (0.000)	–	4.0

Table S6: Predictive and selection performance for $n = 200$, $p = 50$ (exponential baseline). Entries are mean (SE) for IBS and mean for P_{true} and model size.

	$\pi_C = 0.0$			$\pi_C = 0.1$			$\pi_C = 0.3$		
Method	IBS	P_{true}	size	IBS	P_{true}	size	IBS	P_{true}	size
Correlation $\rho = 0.0$									
LASSO-1se	0.259 (0.005)	0.31	5.7	0.129 (0.000)	0.41	5.3	0.145 (0.000)	0.45	5.2
LASSO-aic	0.227 (0.004)	0.00	20.1	0.110 (0.000)	0.01	19.9	0.121 (0.000)	0.00	19.7
LASSO-bic	0.256 (0.004)	0.20	6.3	0.124 (0.000)	0.19	6.3	0.136 (0.000)	0.20	6.4
LASSO-min	0.237 (0.004)	0.00	12.9	0.117 (0.000)	0.00	12.9	0.128 (0.000)	0.00	12.7
Oracle	0.287 (0.003)	–	4.0	0.122 (0.001)	–	4.0	0.136 (0.001)	–	4.0
Correlation $\rho = 0.1$									
LASSO-1se	0.253 (0.005)	0.38	5.5	0.123 (0.000)	0.45	5.2	0.139 (0.000)	0.55	4.9
LASSO-aic	0.210 (0.004)	0.01	18.9	0.106 (0.000)	0.00	19.3	0.117 (0.000)	0.01	19.4
LASSO-bic	0.247 (0.004)	0.25	6.0	0.118 (0.000)	0.23	6.1	0.131 (0.000)	0.23	6.1
LASSO-min	0.231 (0.004)	0.00	12.4	0.112 (0.000)	0.00	12.4	0.124 (0.000)	0.00	12.2
Oracle	0.275 (0.003)	–	4.0	0.117 (0.001)	–	4.0	0.131 (0.001)	–	4.0

Table S7: Predictive and selection performance for $n = 200$, $p = 50$ (Weibull baseline). Entries are mean (SE) for IBS and mean for P_{true} and model size.

	$\pi_C = 0.0$			$\pi_C = 0.1$			$\pi_C = 0.3$		
Method	IBS	P_{true}	size	IBS	P_{true}	size	IBS	P_{true}	size
Correlation $\rho = 0.0$									
LASSO-1se	0.239 (0.004)	0.36	5.7	0.130 (0.000)	0.36	5.5	0.136 (0.000)	0.41	5.3
LASSO-aic	0.199 (0.003)	0.00	20.5	0.112 (0.000)	0.00	20.2	0.114 (0.000)	0.00	20.3
LASSO-bic	0.225 (0.003)	0.18	6.4	0.125 (0.000)	0.17	6.3	0.129 (0.000)	0.19	6.4
LASSO-min	0.213 (0.003)	0.00	12.8	0.119 (0.000)	0.00	12.7	0.122 (0.000)	0.00	12.3
Oracle	0.257 (0.003)	–	4.0	0.123 (0.000)	–	4.0	0.128 (0.000)	–	4.0
Correlation $\rho = 0.1$									
LASSO-1se	0.231 (0.003)	0.41	5.5	0.125 (0.000)	0.45	5.3	0.132 (0.000)	0.50	5.0
LASSO-aic	0.198 (0.003)	0.00	19.4	0.109 (0.000)	0.01	18.7	0.111 (0.000)	0.01	19.3
LASSO-bic	0.218 (0.003)	0.25	6.0	0.121 (0.000)	0.24	5.9	0.125 (0.000)	0.24	6.1
LASSO-min	0.208 (0.003)	0.00	12.5	0.114 (0.000)	0.00	12.3	0.118 (0.000)	0.00	12.2
Oracle	0.249 (0.003)	–	4.0	0.118 (0.000)	–	4.0	0.124 (0.000)	–	4.0

Table S8: Performance measures of the realistic simulation based on METABRIC for $n = 200$. Entries are mean (SE) across Monte Carlo repetitions.

Method	$\pi_C = 0.0$		$\pi_C = 0.1$		$\pi_C = 0.3$	
	C-index	IBS	C-index	IBS	C-index	IBS
LASSO-1se	0.477 (0.001)	0.965 (0.026)	0.499 (0.000)	0.896 (0.026)	0.500 (0.000)	0.744 (0.034)
LASSO-aic	0.433 (0.002)	1.038 (0.032)	0.499 (0.001)	0.900 (0.026)	0.501 (0.001)	0.775 (0.035)
LASSO-bic	0.434 (0.002)	1.038 (0.032)	0.499 (0.001)	0.913 (0.035)	0.501 (0.001)	0.769 (0.035)
LASSO-min	0.433 (0.002)	1.047 (0.035)	0.499 (0.001)	0.900 (0.027)	0.501 (0.001)	0.772 (0.035)
Oracle	0.609 (0.001)	0.761 (0.022)	0.501 (0.001)	0.918 (0.028)	0.501 (0.001)	1.023 (0.030)

Table S9: C-index (mean (SD)) for $n = 200$, $p = 10$ for both exponential and Weibull baseline

ρ	Method	Exponential baseline			Weibull baseline		
		$\pi_C = 0.0$	$\pi_C = 0.1$	$\pi_C = 0.3$	$\pi_C = 0.0$	$\pi_C = 0.1$	$\pi_C = 0.3$
	LASSO-1se	0.781 (0.016)	0.783 (0.017)	0.789 (0.019)	0.781 (0.016)	0.784 (0.017)	0.790 (0.019)
	LASSO-aic	0.783 (0.015)	0.787 (0.016)	0.794 (0.018)	0.783 (0.016)	0.786 (0.017)	0.794 (0.019)
0.0	LASSO-bic	0.782 (0.016)	0.786 (0.016)	0.793 (0.019)	0.782 (0.016)	0.786 (0.017)	0.793 (0.019)
	LASSO-min	0.783 (0.016)	0.786 (0.016)	0.793 (0.018)	0.783 (0.017)	0.785 (0.017)	0.794 (0.019)
	Oracle	0.776 (0.023)	0.778 (0.025)	0.784 (0.028)	0.777 (0.023)	0.779 (0.025)	0.783 (0.028)
	LASSO-1se	0.790 (0.015)	0.794 (0.016)	0.800 (0.018)	0.791 (0.015)	0.793 (0.017)	0.800 (0.019)
	LASSO-aic	0.793 (0.015)	0.795 (0.016)	0.803 (0.018)	0.793 (0.015)	0.796 (0.016)	0.804 (0.019)
0.1	LASSO-bic	0.792 (0.015)	0.795 (0.016)	0.802 (0.018)	0.792 (0.015)	0.795 (0.016)	0.802 (0.019)
	LASSO-min	0.792 (0.015)	0.796 (0.016)	0.803 (0.018)	0.793 (0.015)	0.796 (0.016)	0.802 (0.018)
	Oracle	0.785 (0.023)	0.787 (0.024)	0.794 (0.027)	0.785 (0.023)	0.787 (0.024)	0.792 (0.028)

S3 Additional results data example

S3.1 Selection frequencies for METABRIC data example

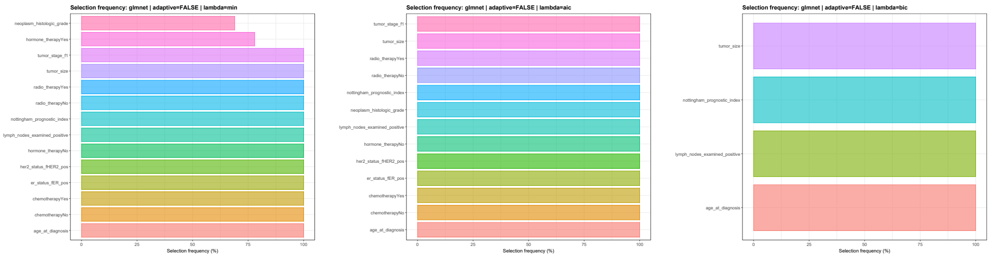

(a) glmnet, $\lambda_{\mathrm{CV,min}}$ (b) glmnet, λ_{AIC} (c) glmnet, λ_{BIC}

Fig. S31: Selection frequencies for the METABRIC data example (glmnet / Lasso). Bars show the percentage of subsamples in which each covariate was selected.

Table S10: C-index (mean (SD)) for $n = 200$, $p = 20$, for both exponential and Weibull baseline

		Exponential baseline			Weibull baseline		
ρ	Method	$\pi_C = 0.0$	$\pi_C = 0.1$	$\pi_C = 0.3$	$\pi_C = 0.0$	$\pi_C = 0.1$	$\pi_C = 0.3$
	LASSO-1se	0.781 (0.015)	0.784 (0.016)	0.789 (0.019)	0.781 (0.015)	0.784 (0.017)	0.790 (0.019)
	LASSO-aic	0.788 (0.015)	0.791 (0.016)	0.799 (0.018)	0.788 (0.016)	0.791 (0.017)	0.799 (0.019)
0.0	LASSO-bic	0.783 (0.016)	0.786 (0.017)	0.795 (0.019)	0.783 (0.016)	0.787 (0.017)	0.794 (0.019)
	LASSO-min	0.786 (0.016)	0.789 (0.016)	0.797 (0.018)	0.786 (0.016)	0.789 (0.017)	0.797 (0.019)
	Oracle	0.776 (0.023)	0.778 (0.024)	0.784 (0.027)	0.776 (0.023)	0.778 (0.024)	0.783 (0.028)
	LASSO-1se	0.791 (0.016)	0.792 (0.016)	0.801 (0.017)	0.791 (0.015)	0.793 (0.017)	0.801 (0.018)
	LASSO-aic	0.797 (0.015)	0.799 (0.016)	0.808 (0.018)	0.796 (0.016)	0.801 (0.016)	0.808 (0.018)
0.1	LASSO-bic	0.793 (0.016)	0.795 (0.016)	0.805 (0.018)	0.794 (0.015)	0.796 (0.016)	0.804 (0.019)
	LASSO-min	0.796 (0.016)	0.798 (0.016)	0.806 (0.018)	0.795 (0.015)	0.798 (0.016)	0.806 (0.019)
	Oracle	0.786 (0.023)	0.788 (0.024)	0.795 (0.027)	0.786 (0.023)	0.788 (0.024)	0.794 (0.027)

Table S11: C-index (mean (SD)) for $n = 200$, $p = 50$ for both exponential and Weibull baseline

		Exponential baseline			Weibull baseline		
ρ	Method	$\pi_C = 0.0$	$\pi_C = 0.1$	$\pi_C = 0.3$	$\pi_C = 0.0$	$\pi_C = 0.1$	$\pi_C = 0.3$
	LASSO-1se	0.782 (0.017)	0.784 (0.017)	0.789 (0.021)	0.781 (0.016)	0.784 (0.017)	0.791 (0.019)
	LASSO-aic	0.797 (0.018)	0.801 (0.018)	0.812 (0.021)	0.797 (0.018)	0.802 (0.019)	0.813 (0.022)
0.0	LASSO-bic	0.784 (0.016)	0.786 (0.017)	0.794 (0.020)	0.784 (0.016)	0.788 (0.017)	0.795 (0.020)
	LASSO-min	0.790 (0.016)	0.793 (0.017)	0.803 (0.018)	0.790 (0.015)	0.793 (0.017)	0.802 (0.020)
	Oracle	0.775 (0.023)	0.777 (0.025)	0.784 (0.028)	0.775 (0.024)	0.777 (0.025)	0.783 (0.028)
	LASSO-1se	0.792 (0.015)	0.795 (0.016)	0.800 (0.019)	0.791 (0.015)	0.794 (0.016)	0.801 (0.019)
	LASSO-aic	0.805 (0.017)	0.809 (0.018)	0.819 (0.020)	0.806 (0.017)	0.808 (0.018)	0.820 (0.021)
0.1	LASSO-bic	0.793 (0.015)	0.796 (0.017)	0.804 (0.018)	0.793 (0.015)	0.795 (0.016)	0.805 (0.019)
	LASSO-min	0.798 (0.016)	0.802 (0.016)	0.811 (0.018)	0.799 (0.016)	0.802 (0.017)	0.811 (0.018)
	Oracle	0.785 (0.023)	0.787 (0.024)	0.794 (0.027)	0.785 (0.024)	0.788 (0.025)	0.794 (0.028)

Table S12: METABRIC ($n = 200$): C-index (mean (SD))

Method	$\pi_C = 0.0$	$\pi_C = 0.1$	$\pi_C = 0.3$
LASSO-1se	0.477 (0.037)	0.499 (0.012)	0.500 (0.012)
LASSO-aic	0.433 (0.051)	0.499 (0.020)	0.501 (0.020)
LASSO-bic	0.434 (0.051)	0.499 (0.020)	0.501 (0.021)
LASSO-min	0.433 (0.051)	0.499 (0.020)	0.501 (0.020)
Oracle	0.609 (0.038)	0.501 (0.036)	0.501 (0.037)

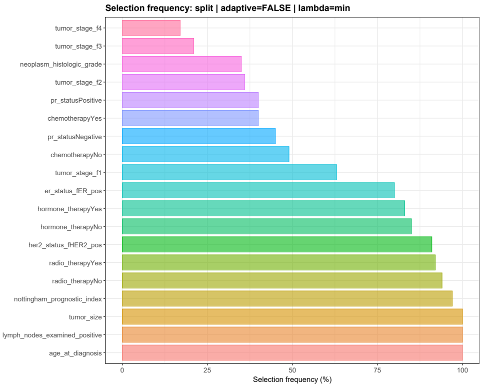

(a) Sample splitting, $\lambda_{\mathrm{CV,min}}$

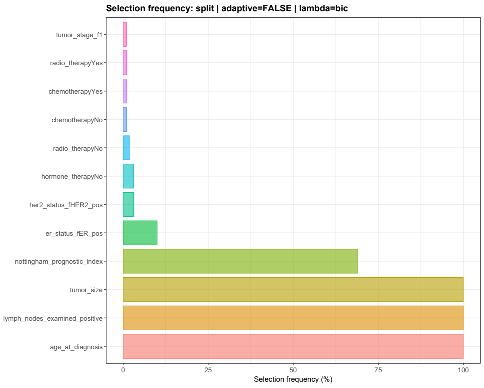

(b) Sample splitting, λ_{BIC}

Fig. S32: Selection frequencies for the METABRIC data example (sample splitting). Bars show the percentage of subsamples in which each covariate was selected.

S3.2 Point estimates and 90% post-selection confidence intervals for regression coefficients

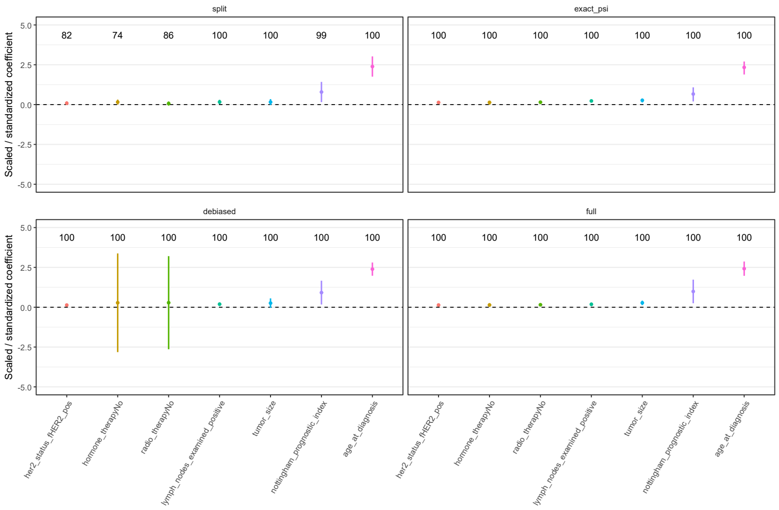

Fig. S33: Real data example (METABRIC): point estimates and 90% post-selection confidence intervals for regression coefficients obtained with different inference methods. Results are shown for the tuning choice λ_{AIC}. Coefficients are displayed on the original scale and ordered by increasing standardized effect size. Numbers above the panels indicate selection frequencies (in %) across 100 subsamples.

References

[1] Tibshirani, R.: Regression shrinkage and selection via the Lasso. Journal of the Royal Statistical Society Series B: Statistical Methodology **58**(1), 267–288 (1996) https://doi.org/10.1111/j.2517-6161.1996.tb02080.x

[2] Zou, H.: The Adaptive Lasso and its oracle properties. Journal of the American Statistical Association **101**(476), 1418–1429 (2006) https://doi.org/10.1198/016214506000000735

[3] Berk, R., Brown, L., Buja, A., Zhang, K., Zhao, L.: Valid post-selection inference. The Annals of Statistics **41**(2), 802–837 (2013) https://doi.org/10.1214/12-AOS1077

[4] Lee, J.D., Sun, D.L., Sun, Y., Taylor, J.E.: Exact post-selection inference, with application to the Lasso. The Annals of Statistics **44**(3), 907–927 (2016) https://doi.org/10.1214/15-AOS1371

[5] Andersen, P.K., Borgan, Ø., Gill, R.D., Keiding, N.: Model specification and censoring. In: Statistical Models Based on Counting Processes, pp. 121–175. Springer, New York (1993). Chap. 3

[6] Tibshirani, R.: The Lasso method for variable selection in the Cox model. Statistics in Medicine **16**(4), 385–395 (1997) https://doi.org/10.1002/(sici)1097-0258(19970228)16:4⟨385::aid-sim380⟩3.0.co;2-3

[7] Zhang, H.H., Lu, W.: Adaptive Lasso for Cox's proportional hazards model. Biometrika **94**(3), 691–703 (2007) https://doi.org/10.1093/biomet/asm037

[8] Cox, D.R.: A note on data-splitting for the evaluation of significance levels. Biometrika **62**(2), 441–444 (1975) https://doi.org/10.2307/2335385

[9] Taylor, J., Tibshirani, R.J.: Statistical learning and selective inference. Proceedings of the National Academy of Sciences **112**(25), 7629–7634 (2015) https://doi.org/10.1073/pnas.1507583112

[10] Yu, Y., Bradic, J., Samworth, R.J.: Confidence intervals for high-dimensional Cox models. Statistica Sinica **31**(1), 243–267 (2021) https://doi.org/10.1111/rssb.12026

[11] Kong, S., Yu, Z., Zhang, X., Cheng, G.: High-dimensional robust inference for Cox regression models using desparsified Lasso. Scandinavian Journal of Statistics **48**(3), 1068–1095 (2021) https://doi.org/10.1111/sjos.12543

[12] Fithian, W., Sun, D., Taylor, J.: Optimal Inference After Model Selection (2017). https://arxiv.org/abs/1410.2597

[13] Taylor, J., Tibshirani, R.: Post-selection inference for ℓ_1-penalized likelihood models. Canadian Journal of Statistics **46**(1), 41–61 (2018) https://doi.org/10.1002/cjs.11313

[14] Zhang, C.-H., Zhang, S.S.: Confidence intervals for low dimensional parameters in high dimensional linear models. Journal of the Royal Statistical Society Series B: Statistical Methodology **76**(1), 217–242 (2014) https://doi.org/10.1111/rssb.12026

[15] van de Geer, S., Bühlmann, P., Ritov, Y., Dezeure, R.: On asymptotically optimal confidence regions and tests for high-dimensional models. The Annals of Statistics **42**(3), 1166–1202 (2014) https://doi.org/10.1214/14-AOS1221

[16] Kammer, M., Dunkler, D., Michiels, S., Heinze, G.: Evaluating methods for Lasso selective inference in biomedical research: a comparative simulation study. BMC Medical Research Methodology **22**(1), 206 (2022) https://doi.org/10.1186/s12874-022-01681-y

[17] Cox, D.R.: Regression models and life-tables. Journal of the Royal Statistical Society: Series B (Methodological) **34**(2), 187–220 (1972) https://doi.org/10.1111/j.2517-6161.1972.tb00899.x

[18] Fan, J., Li, R.: Variable selection for Cox's proportional hazards model and frailty model. The Annals of Statistics **30**(1), 74–99 (2002) https://doi.org/10.1214/aos/1015362185

[19] Tang, Z., Shen, Y., Zhang, X., Yi, N.: The spike-and-slab lasso cox model for survival prediction and associated genes detection. Bioinformatics **33**(18), 2799–2807 (2017) https://doi.org/10.1093/bioinformatics/btx300

[20] Wang, L., Chang, Y., Ma, J., Qu, W., Li, Y.: Identifying high-risk candidates for prolonging progression-free survival in primary gastric carcinoma subject to "double invasion": an analytical approach utilizing lasso-cox regression. BMC Cancer **25**(1), 381 (2025) https://doi.org/10.1186/s12885-025-12054-5

[21] Wainwright, M.J.: Sharp thresholds for high-dimensional and noisy sparsity recovery using ℓ_1-constrained quadratic programming (lasso). IEEE Transactions on Information Theory **55**(5), 2183–2202 (2009) https://doi.org/10.1109/TIT.2009.2016018

[22] Hastie, T., Tibshirani, R., Wainwright, M.: Statistical Learning with Sparsity: The Lasso and Generalizations. Chapman & Hall/CRC, Boca Raton, FL (2015). Chapman & Hall/CRC Monographs on Statistics and Applied Probability

[23] Leeb, H., Pötscher, B.M.: Model selection and inference: Facts and fiction. Econometric Theory **21**(1), 21–59 (2005) https://doi.org/10.1017/S0266466605050036

[24] Leeb, H., Pötscher, B.M.: Can one estimate the conditional distribution of post-model-selection estimators? The Annals of Statistics **34**(5), 2554–2591 (2006) https://doi.org/10.1214/009053606000000821

[25] Therneau, T.M., Grambsch, P.M.: Modeling Survival Data: Extending the Cox Model. Springer, New York (2000)

[26] Gail, M.H., Wieand, S., Piantadosi, S.: Biased estimates of treatment effect in randomized experiments with nonlinear regressions and omitted covariates. Biometrika **71**(3), 431–444 (1984) https://doi.org/10.1093/biomet/71.3.431

[27] Aalen, O.O., Cook, R.J., Røysland, K.: Does cox analysis of a randomized survival study yield a causal treatment effect? Lifetime Data Analysis **21**(4), 579–593 (2015) https://doi.org/10.1007/s10985-015-9335-y

[28] Daniel, R., Zhang, J., Farewell, D.: Making apples from oranges: Comparing noncollapsible effect estimators and their standard errors after adjustment for different covariate sets. Biometrical Journal **63**(3), 528–557 (2021) https://doi.org/10.1002/bimj.201900297

[29] Tian, X., Taylor, J.: Selective inference with a randomized response. The Annals of Statistics **46**(2), 679–710 (2018) https://doi.org/10.1214/17-AOS1564

[30] Meinshausen, N., Meier, L., Bühlmann, P.: P-values for high-dimensional regression. Journal of the American Statistical Association **104**(488), 1671–1681 (2009) https://doi.org/10.1198/jasa.2009.tm08647

[31] Xia, L., Nan, B., Li, Y.: Statistical inference for cox proportional hazards models with a diverging number of covariates. Scandinavian Journal of Statistics **50**(2), 550–571 (2023) https://doi.org/10.1111/sjos.12595

[32] Morris, T.P., White, I.R., Crowther, M.J.: Using simulation studies to evaluate statistical methods. Statistics in Medicine **38**(11), 2074–2102 (2019) https://doi.org/10.1002/sim.8086

[33] Ramos, P.L., Guzman, D.C.F., Mota, A.L., Saavedra, D.A., Rodrigues, F.A., Louzada, F.: Sampling with censored data: a practical guide. Journal of Statistical Computation and Simulation **94**(18), 4072–4106 (2024) https://doi.org/10.1080/00949655.2024.2409379

[34] Andersen, P.K., Gill, R.D.: Cox's regression model for counting processes: A large sample study. Annals of Statistics **10**(4), 1100–1120 (1982) https://doi.org/10.1214/aos/1176345976

[35] Curtis, C., Shah, S.P., Chin, S.-F., Turashvili, G., Rueda, O.M., Dunning, M.J., Speed, D., Lynch, A.G., Samarajiwa, S., Yuan, Y., Gräf, S., Ha, G., Haffari, G., Bashashati, A., Russell, R., McKinney, S., Langerød, A., Green, A., Provenzano,

E., Wishart, G., Pinder, S., Watson, P., Markowetz, F., Murphy, L., Ellis, I., Purushotham, A., Børresen-Dale, A.-L., Brenton, J.D., Tavaré, S., Aparicio, S., Caldas, C.: The genomic and transcriptomic architecture of 2,000 breast tumours reveals novel subgroups. Nature **486**(7403), 346–352 (2012) https://doi.org/10.1038/nature10983

[36] Sauer, C., Lange, F.J.D., Thurow, M., Dormuth, I., Boulesteix, A.-L.: Statistical parametric simulation studies based on real data (2025). https://arxiv.org/abs/2504.04864

[37] Tibshirani, R., Tibshirani, R., Taylor, J., Loftus, J., Reid, S., Markovic, J.: selectiveInference: Tools for Post-Selection Inference. (2019). R package version 1.2.5. https://CRAN.R-project.org/package=selectiveInference

[38] Hastie, T., Tibshirani, R., Friedman, J.: The Elements of Statistical Learning, 2nd edn. Springer, New York, NY, USA (2009). https://doi.org/10.1007/978-0-387-84858-7

[39] Akaike, H.: A new look at the statistical model identification. IEEE Transactions on Automatic Control **19**(6), 716–723 (1974) https://doi.org/10.1109/TAC.1974.1100705

[40] Schwarz, G.: Estimating the dimension of a model. The Annals of Statistics **6**(2), 461–464 (1978) https://doi.org/10.1214/aos/1176344136

[41] Fan, J., Li, R.: Variable selection via nonconcave penalized likelihood and its oracle properties. Journal of the American Statistical Association **96**(456), 1348–1360 (2001) https://doi.org/10.1198/016214501753382273

[42] Blanche, P., Kattan, M.W., Gerds, T.A.: The c-index is not proper for the evaluation of t-year predicted risks. Biostatistics **20**(2), 347–357 (2019) https://doi.org/10.1093/biostatistics/kxy006

[43] Gerds, T.A., Schumacher, M.: Consistent estimation of the expected brier score in general survival models with right-censored event times. Biometrical Journal **48**(6), 1029–1040 (2006) https://doi.org/10.1002/bimj.200610301

[44] Therneau, T.M.: A Package for Survival Analysis in R. (2024). R package version 3.7-0. https://CRAN.R-project.org/package=survival

[45] Friedman, J., Hastie, T., Tibshirani, R., Narasimhan, B., Tay, K., Simon, N., Qian, J.: Glmnet: Lasso and Elastic-Net Regularized Generalized Linear Models. (2021). R package version 4.1-1. https://CRAN.R-project.org/package=glmnet

[46] Friedman, J., Hastie, T., Tibshirani, R.: Regularization paths for generalized linear models via coordinate descent. Journal of Statistical Software **33**(1), 1–22 (2010) https://doi.org/10.18637/jss.v033.i01

[47] Simon, N., Friedman, J., Hastie, T., Tibshirani, R.: Regularization paths for cox's proportional hazards model via coordinate descent. Journal of Statistical Software **39**(5), 1–13 (2011) https://doi.org/10.18637/jss.v039.i05

[48] Vovk, V., Wang, R.: E-values: Calibration, combination, and applications. The Annals of Statistics **49**(3), 1736–1754 (2021) https://doi.org/10.1214/20-AOS2020

[49] Xu, Z., Wang, R., Ramdas, A.: Post-selection inference for e-value based confidence intervals. Electronic Journal of Statistics **18**(1), 2292–2338 (2024) https://doi.org/10.1214/24-EJS2253
